# Organoid cultures from normal and cancer-prone human breast tissues preserve complex epithelial lineages

**DOI:** 10.1038/s41467-020-15548-7

**Published:** 2020-04-06

**Authors:** Jennifer M. Rosenbluth, Ron C. J. Schackmann, G. Kenneth Gray, Laura M. Selfors, Carman Man-Chung Li, Mackenzie Boedicker, Hendrik J. Kuiken, Andrea Richardson, Jane Brock, Judy Garber, Deborah Dillon, Norman Sachs, Hans Clevers, Joan S. Brugge

**Affiliations:** 1 Department of Cell Biology, Harvard Medical School, 240 Longwood Ave., Boston, MA 02115 USA; 2 Department of Pathology, Brigham & Women’s Hospital, 75 Francis St, Boston, MA 02115 USA; 3 Department of Medical Oncology, Dana-Farber Cancer Institute, 450 Brookline Ave, Boston, MA 02115 USA; 4 Hubrecht Institute, Uppsalalaan 8, 3584 CT Utrecht, The Netherlands

**Keywords:** Biological models, Mammary stem cells, Oncogenesis

## Abstract

Recently, organoid technology has been used to generate a large repository of breast cancer organoids. Here we present an extensive evaluation of the ability of organoid culture technology to preserve complex stem/progenitor and differentiated cell types via long-term propagation of normal human mammary tissues. Basal/stem and luminal progenitor cells can differentiate in culture to generate mature basal and luminal cell types, including ER+ cells that have been challenging to maintain in culture. Cells associated with increased cancer risk can also be propagated. Single-cell analyses of matched organoid cultures and native tissues by mass cytometry for 38 markers provide a higher resolution representation of the multiple mammary epithelial cell types in the organoids, and demonstrate that protein expression patterns of the tissue of origin can be preserved in culture. These studies indicate that organoid cultures provide a valuable platform for studies of mammary differentiation, transformation, and breast cancer risk.

## Abstract (web summary)

Organoid technology has enabled the generation of several breast cancer organoids. Here, the authors combine propagation of normal human mammary tissues with mass cytometry to evaluate the ability of organoid culture technologies to preserve stem cells and differentiated cell types.

## Introduction

The ability to culture mammary epithelial cells has contributed to ground-breaking discoveries that have provided important insights into the regulatory mechanisms of normal cell behavior and tumorigenic transformation^1–3^. However, propagation of all of the major lineages within the breast for an extended period of time has remained challenging. This has limited investigation into the early stages of oncogenic transformation of different subtypes of breast cancer (BC), as many culture technologies are unable to preserve the progenitor cells that may be the specific cancer cell of origin. A technique for long-term culture of BC organoids has recently been developed^4^. The extent to which normal human mammary epithelial cells (HMECs) across multiple lineages can be cultured indefinitely in this system has not been established.

Mammary epithelial cells are broadly grouped into two categories, luminal and basal, based on their location within a bilayered breast epithelium. The majority of basal cells are differentiated, contractile myoepithelial cells^5^; however, a subset of cells that express basal surface markers (CD49^+^EpCAM^−^) have been shown to have the capability to generate both basal and luminal cells in colony forming assays in vitro^6^ and in vivo (following transplantation into mouse mammary glands^7,8^ or kidney capsules^9^), thus, displaying properties of tissue-specific adult progenitor cells. Luminal cells include mature luminal cells that function in hormone sensing and milk production, as well as luminal progenitor cells which are progenitor-like but committed to the luminal lineage^5^. Recent studies of murine models as well as human tissues have suggested far greater complexity of mammary epithelial hierarchy, and have also identified specific differences in the composition of these epithelial compartments associated with pregnancy, with aging, and with inherited mutations in BC predisposition genes^10–14^. These studies and others suggest a high level of complexity within the mammary epithelium, with one or more cell types contributing to the development of BC.

We report here the characterization of organoid cultures of epithelial cells derived from histologically normal breast tissue, either from women who underwent an elective reduction mammoplasty or from women with an inherited mutation in a cancer syndrome gene (prophylactic mastectomy). Organoids can be passaged continuously in culture, preserve all of the major mammary epithelial lineages, and retain expression patterns of mammary markers from the breast tissue of origin as determined by single-cell mass cytometry (cytometry by time of flight (CyTOF)). Organoid culture also recapitulates the expansion of the luminal progenitor population associated with *BRCA1* heterozygosity. Thus, organoid technology allows the growth and characterization of multiple normal mammary epithelial cell lineages in a single culture, which will enable a greater understanding of the genesis of different BC subtypes.

## Results

### Propagation of normal human mammary organoids

We successfully established 79 organoid cultures from normal human mammary tissues obtained either from reduction mammoplasties (performed to reduce breast size) or from prophylactic mastectomies (performed to prevent BC) using the culture conditions described previously^4^. In all cases, normal histology of the originating tissue was confirmed upon review by a breast pathologist (D.D.). The rate of establishment of organoid cultures was high, with an efficiency of 95%. As with other organoid systems^15^, cultures could be propagated long term, with the longest organoid culture passaged for >16 months. Organoids were typically dissociated and passaged every 2–4 weeks.

**Fig. 1:**
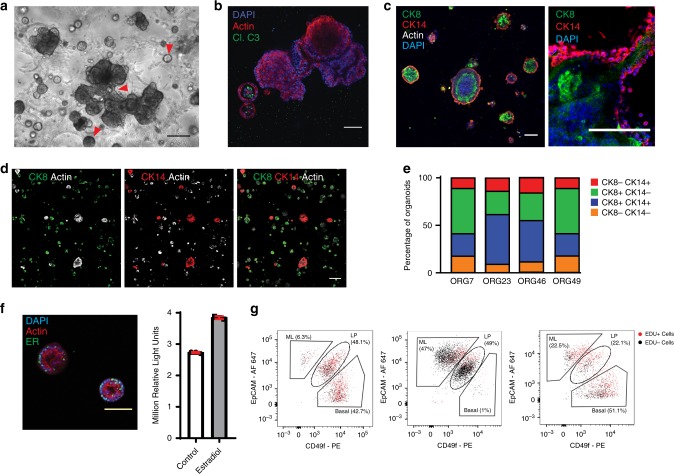
Human mammary epithelium can be propagated without immortalization as organoid cultures. **a** Human mammary tissue was digested and cultured in BC organoid medium as previously described^4^. A culture exhibiting multiple organoid structure types (indicated by red arrowheads, representative of *n* = 79) was imaged by phase contrast microscopy, scale bar = 100 µm. **b**, **c** Organoids were stained as indicated for actin (red) and cleaved caspase 3 (green) to demonstrate clearance of a lumen, or stained for CK8 (green), CK14 (red), and actin (white), and counter-stained with DAPI (blue), *n* = 4, scale bar = 100 µm. Organoids were imaged by confocal microscopy. **d** Low magnification confocal images for a representative culture are shown, *n* = 4, scale bar = 100 µm. **e** Quantification of organoids positive for CK8 and/or CK14 in each of four normal mammary cultures. **f** Organoids were stained for ERα (green), actin (red), and DAPI (blue) and imaged by confocal microscopy (left panel, scale bar = 100 µm). Organoids were treated with 0.5 ng/ml estradiol for 7 days, and organoid viability was assayed using Cell-Titer-Glo (right panel). Mean and standard deviation are shown (ORG9, *n* = 3). **g** Three representative organoid cultures (ORG45, ORG9, and ORG24) were stained with EpCAM and CD49f, and these markers as well as EDU incorporation were measured using flow cytometry.

Organoids of several tissue types have been found to exhibit a single defining morphology that resembles the histology of the tissue of origin, such as the intestinal crypt^16^. In contrast, we found that mammary epithelial cells self-organized into multiple different structure types in organoid culture (Fig. 1a, b). The majority of structures were acinar-type and had a lumen, which was either isolated or associated with a budding organoid. Solid spheres were also present, in addition to branching duct-like structures. Branching or budding structures were present in 1 out of 102 organoids (*n* = 3 cultures, Supplementary Table 1). The latter were noted during early passages but became less frequent with repeated dissociation and extended growth. Organoids were stained for the luminal marker cytokeratin 8 (CK8), and the basal marker cytokeratin 14 (CK14), and were found to be comprised of luminal cells, basal cells or a mixture of both cell types (Fig. 1c–e), indicating that both basal and luminal cells are maintained in these cultures.

This culture method was particularly notable for the efficient propagation of luminal cells. This was evident based on luminal cytokeratin expression by immunofluorescence, the identification of EpCAM-high cells by flow cytometry, and the generation of acinar-type luminal organoid structures. A subset of mammary organoids maintained estrogen receptor alpha (ERα) expression in culture, and grew in response to estradiol treatment (Fig. 1f and Supplementary Fig. 1a). An average of 34% of organoids within a culture contained at least one cell that was positive for ER (*n* = 5 cultures). When counted per cell instead of per organoid, an average of 10% of all cells within a culture were positive for ER (*n* = 5 cultures) (Supplementary Table 2).

Flow cytometry analysis of the organoid cultures using the markers EpCAM and CD49f demonstrated that mature luminal (EpCAM^+^ CD49f^−^), luminal progenitor (EpCAM^+^ CD49f^+^), and basal/stem (EpCAM^−^ CD49f^+^) cells were all maintained in culture (see three representative examples in Fig. 1g).

### Mammary lineages form distinct organoid morphologies

**Fig. 2:**
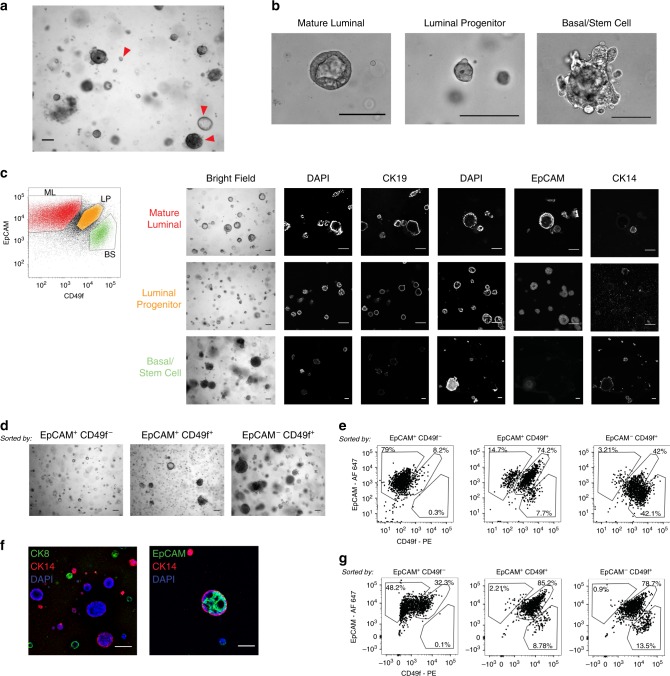
Breast epithelial cell types form distinct structures in organoid culture. **a** Bright field microscopy image of a representative organoid culture demonstrating multiple structure types, highlighted by red arrowheads. **b** Representative examples of organoid morphology for each of the indicated mammary cell lineages. **c** Cells from an organoid culture (representative of *n* = 9 cultures) were stained for EpCAM and CD49f, and sorted using FACS into mature luminal (ML, red), luminal progenitor (LP, orange), and basal/stem cell (BS, green) populations. Cells were recultured as organoids, and then assessed by bright field microscopy (*n* = 9), or confocal microscopy after staining for the indicated markers (column 1, columns 2–3, and columns 4–6 are from separate images, *n* = 3). Scale bars = 100 µm. **d**, **e** Cells of the indicated types were recultured after sorting for >6 weeks, then imaged by bright field microscopy in **d**, (representative of *n* = 9, scale bar = 100 µm), or analyzed for EpCAM and CD49f expression by flow cytometry in **e**. Cultures with luminal progenitor cells that differentiated to generate other mammary cell types were ORG7, ORG43, ORG46, ORG48, ORG49, and ORG51. ORG41, ORG42, and ORG50 had luminal progenitor cells that did not differentiate to generate other mammary cell types. **f** Luminal progenitor cells (EpCAM^+^ CD49f^+^) sorted from two different organoid cultures were recultured as organoids, stained for CK8 (green) or EpCAM (green), CK14 (red), and with DAPI (blue), and imaged by confocal microscopy (representative of *n* = 6, scale bar = 100 µm). **g** Cells were double-sorted for EpCAM and CD49f, then re-grown and assayed as in **e**.

Morphologic assessment of individual organoids within a culture showed that they exhibit diverse structure types; upon subsequent analysis, each morphology could be matched to a distinct mammary cell lineage (Fig. 2a, b). Cells from organoids were sorted by FACS using EpCAM and CD49f into mature luminal (EpCAM^+^ CD49f^−^), luminal progenitor (EpCAM^+^ CD49f^+^), and basal/stem cell (EpCAM^−^ CD49f^+^) subtypes^6,10,17,18^, and then recultured under organoid conditions. Sorted cells expressed basal and luminal markers as expected (Fig. 2c). Mature luminal cells formed acini with a lumen, whereas luminal progenitor cells formed smaller spheres with a smaller lumen, and the latter organoid type expressed known markers of LP cells (Fig. 2b, c and Supplementary Fig. 1b, c). Basal/stem cells formed large disorganized spheres that could exhibit luminal budding or branching outgrowths (Fig. 2b, c).

These structure types are similar to those generated by other three-dimensional mammary culture methods, reflective of the inherent ability of normal mammary cells to self-organize into duct or lobule-like structures when exposed to basement membrane extract^19–22^. However, the organoids that we generated were notable in that multiple structures and cell types coexisted within a single culture, and the diversity could be recapitulated even after repeated dissociation and passaging of organoids. All cell types could form organoids. Although basal/stem cells formed larger organoids, their overall organoid-forming efficiency was lower than the other cell types. Organoid-forming efficiency was calculated as 1 in 16 mature luminal cells, 1 in 10 luminal progenitor cells, and 1 in 117 basal/stem cells (*n* ≥ 3 cultures assessed for each) (Supplementary Table 3).

To assess their growth and differentiation in culture, cells sorted by EpCAM and CD49f were subsequently followed in organoid culture over the course of at least 6 weeks. Whereas EpCAM^+^ CD49f^−^ mature luminal cells generated predominantly acinar structures that remained EpCAM^+^, EpCAM^−^ CD49f^+^ basal/stem cells generated more diverse structure types including basal, luminal progenitor, and mature luminal cell types (Fig. 2d, e). Interestingly, sorted EpCAM^+^ CD49f^+^ luminal progenitor cells were able to generate both mature luminal as well as basal cells (Fig. 2d, e, six of nine cultures tested). Immunofluorescent staining confirmed basal and luminal marker expression in organoids derived from single luminal progenitor cells (Fig. 2f). These results could be recapitulated after double-sorting of cells to increase purity (Fig. 2g). The ability of luminal progenitors to generate both luminal and basal cells in vitro and in vivo (in mammary gland reconstitution experiments) has been previously reported^23,24^.

For three out of nine cases, the sorted luminal progenitor cells did not differentiate in culture into either the luminal or basal lineages (percentage of cells that were luminal progenitor after sorting and reculturing was 95.2, 97.4, and 99.6%). In two of these cases, the parental unsorted cultures were predominantly composed of luminal progenitor-type organoids based on morphology (percentage of cells that were luminal progenitor was 96.6%, 30.6%, and 87.4% respectively). It is possible that the mature luminal and basal cell types are not present in some cultures because the luminal progenitor cells are unable to differentiate into other cell types.

### Organoids preserve complex mammary epithelial lineages

In order to obtain a higher resolution view of complex mammary cell types in organoid culture, we utilized mass cytometry, or CyTOF. This technique allowed us to measure the levels of 38 proteins on a single-cell level, as compared with flow cytometry where typically only 2–4 proteins are measured^25,26^. We generated a unique panel of 38 metal-tagged antibodies recognizing proteins associated with mammary cell function, lineage, and tumorigenesis (Supplementary Fig. 2), in order to delineate mammary-specific protein expression patterns on individual cells in the organoid cultures.

Mass cytometry was performed on a panel of 12 organoid cultures derived from normal mammary tissues of patients of different ages (19–58 years old), parity (G0–G4), and inherited mutation status (none, *BRCA1*, *BRCA2*, *TP53*, *ATM*, and *PALB2*). As mentioned, all tissues were confirmed to be histologically normal. The majority of patients had no prior BC history; in two patients with inherited mutations in cancer predisposition genes and a history of prior BC, we also confirmed that the cells in organoid culture were diploid (Supplementary Fig. 3). To minimize stromal contamination, cultures were analyzed after passage 4. Stromal cells can persist in culture during early passages, but by passage 4 there were no stromal cells remaining in 11 out of 12 cultures (described below), with only a small number of residual fibroblasts present in one culture.

**Fig. 3:**
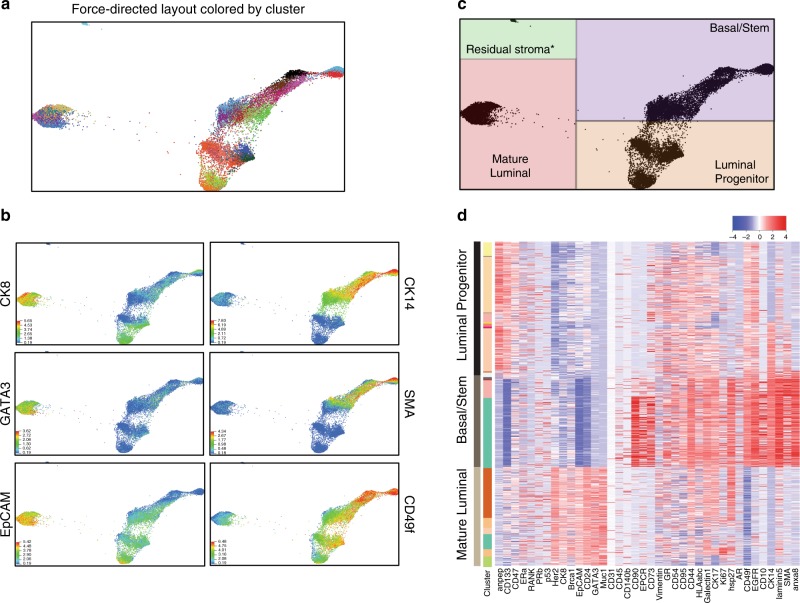
Mass cytometry assessment of mammary cell subtypes in normal breast organoids. **a** Force-directed layout depicting CyTOF analysis of 12 normal mammary organoid cultures. Each dot represents one cell and is colored by X-shift-defined cluster. **b** The expression levels of markers used to define major mammary populations are shown (warmer colors = higher expression levels). **c** Delineation of major mammary lineages on the force-directed layout, as determined by marker expression in **b**. **d** Heat map showing the expression levels of the indicated markers in the cells from a representative organoid culture (red = high expression, blue = low expression). The 38 markers in the CyTOF panel are shown on the *x*-axis, and individual cells on the *y*-axis are ordered based on X-shift clustering.

CyTOF results were analyzed using the X-shift clustering algorithm to find groups or clusters of similar cells, based on the expression levels of the 38 mammary markers. Clusters were then displayed by force-directed layout (Fig. 3a). As described elsewhere^27^, here each dot represents one cell and the proximity of cells as graphed corresponds to the similarity of their protein expression patterns. X-shift analysis defined 29 clusters of epithelial cells in the organoid cultures, representing distinct cell types as well as differences in protein expression within cell lineages and between patients (Fig. 3a). Assessment of basal and luminal markers again confirmed that the major epithelial subtypes were maintained in culture (Fig. 3b–d and Supplementary Fig. 4), with minimal stromal contamination in one culture (121 cells, Fig. 3c). Importantly, we confirmed that BRCA1 and p53 levels were not driving the delineation of the major clusters, even though some of these patients harbored inherited mutations in these genes; re-running the X-shift algorithm without BRCA1 and p53 reproduced nearly identical clustering pattern to the first analysis (Supplementary Fig. 5).

There were several notable findings in the CyTOF analysis of this large set of organoid culture samples, as shown by cluster-based heat map in Fig. 3d and Supplementary Fig. 4, with additional examples shown by force-directed layout in Supplementary Fig. 6. Mature luminal cells in the organoids maintained expression of proteins that are characteristic of their counterparts in primary tissues, e.g., CK8, MUC1, EPCAM, CD24, HER2, and variable expression of ERα^11,28^. PR expression levels in organoids were also variable but generally low. In addition, it has previously been reported that BRCA1 is expressed at the highest level in mature luminal cells, with intermediate expression in luminal progenitor cells and the lowest level of expression in basal/stem cells, and this pattern was well maintained in the organoids^28,29^. Luminal progenitors were distinguished by higher expression of CD133^11^, and basal cells expressed CD49f, and variably expressed SMA, EGFR, CD90, and CD10^30^. In addition, a small (<1%) population of EPCR (also known as PROCR)-expressing cells could be identified within the basal population. PROCR has been shown to mark a rare population of basal multipotent stem cells in the murine mammary gland^31^, and has been shown to be associated with expression of stemness-associated transcription factors and higher mammosphere-forming ability in human mammary cells^32^.

In order to more directly compare protein expression in the organoid cultures to the tissues from which they were derived, four breast tissues were used to generate freshly dissociated single cells that were immediately paraformaldehyde fixed and frozen for CyTOF, as well as organoid cultures that were maintained for four passages, and then dissociated into single cells and prepared for CyTOF. The tissues and the matching organoid cultures were analyzed by CyTOF in the same batch, using our mammary-specific antibody panel. For comparison, one tissue was cultured as HMECs in a canonical two-dimensional culture format, and again the matching HMECs were run in the same batch as cells isolated directly from the tissue of origin.

**Fig. 4:**
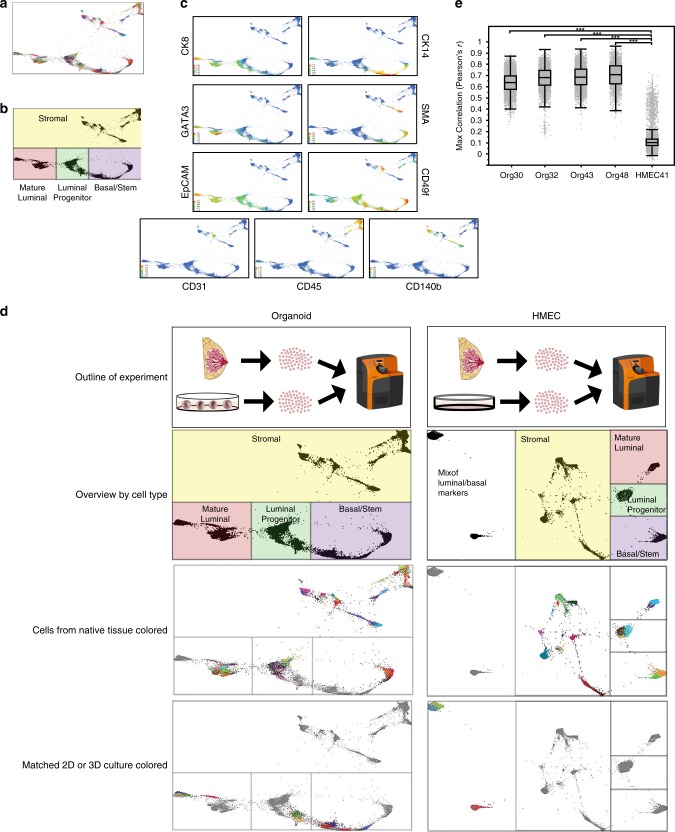
Complex mammary-specific protein expression patterns from the tissue of origin are maintained in culture. Four normal mammary tissues were digested to single cells and fixed with paraformaldehyde. In parallel, organoid cultures were generated from each of the tissues, passaged four times, digested to single cells, and fixed. Protein expression levels of 38 markers associated with mammary tumorigenesis and development were analyzed at the single-cell level in the organoid cultures and the matched tissues of origin using cytometry by time of flight (CyTOF). **a** Force-directed layout of organoid cultures and matched tissues of origin, where each dot represents one cell, and the closeness in space is related to their similarity in terms of protein expression patterns. Cells are colored by X-shift-defined clusters. **b** Delineation of the major mammary populations on the force-directed layout based on expression of standard protein markers for these cell types. **c** Examples of lineage-specific markers used to determine the mammary populations shown in **b**, including luminal (CK8 and EpCAM), mature luminal (GATA3), and basal/stem (CK14, SMA, and CD49f), as well as stromal markers (CD31, CD45, and CD140b) (warmer colors = higher expression levels). **d** For comparison, a normal mammary tissue was digested to single cells and fixed, and in parallel a two-dimensional culture of human mammary epithelial cells (HMECs, cultured in Lonza MEGM) was generated and passaged two times. HMECs and the matching tissue of origin were also analyzed using CyTOF as in **a**. The experimental schematic, delineation of cell types on force-directed layout, and cells from the tissue or cells from the culture are highlighted in the different panels for both the organoids (left column) and the HMECs (right column). **e** Correlation between the protein expression profiles of each HMEC or organoid cell and expression signatures derived from the major epithelial clusters in matched primary tissue. Box plots (center line, median; box limits, upper and lower quartiles; whiskers, 1.5× interquartile range) show the maximum *r* value of each cell to the major epithelial clusters, stratified by sample. Statistical significance was assessed by two-sided Mann–Whitney test (***p < 2.2e−16).

X-shift defined 70 clusters in the four pairs of matched tissues and organoids; 44 of these were epithelial clusters (Fig. 4a). Using standard markers to define stromal cells, as well as luminal and basal epithelial cells, we confirmed that the major mammary epithelial cell types were preserved in culture (Fig. 4b–d). Organoid cultures retained a remarkable degree of similarity to the tissues from which they were derived, as depicted by closeness in space on force-directed layout, as well as X-shift-defined clustering and heat map representation of protein expression (Fig. 4d, left panel, and Supplementary Fig. 7a). In contrast to the organoids, HMECs grown for only two passages in standard two-dimensional culture using Lonza MEGM medium diverged significantly from the primary tissues, lost most luminal cells, and displayed a protein expression pattern with mixed basal and luminal features that did not resemble any cells from the primary tissue (Fig. 4d, right panels, and Supplementary Fig. 7b).

We carried out a statistical comparison of the protein expression profiles of each cell in the HMEC and organoid cultures to signatures derived from the major epithelial types in matched primary tissue. Cells from the organoid cultures demonstrated significantly stronger correlation to primary tissue-derived epithelial expression signatures than cells from the HMEC culture (Fig. 4e).

**Fig. 5:**
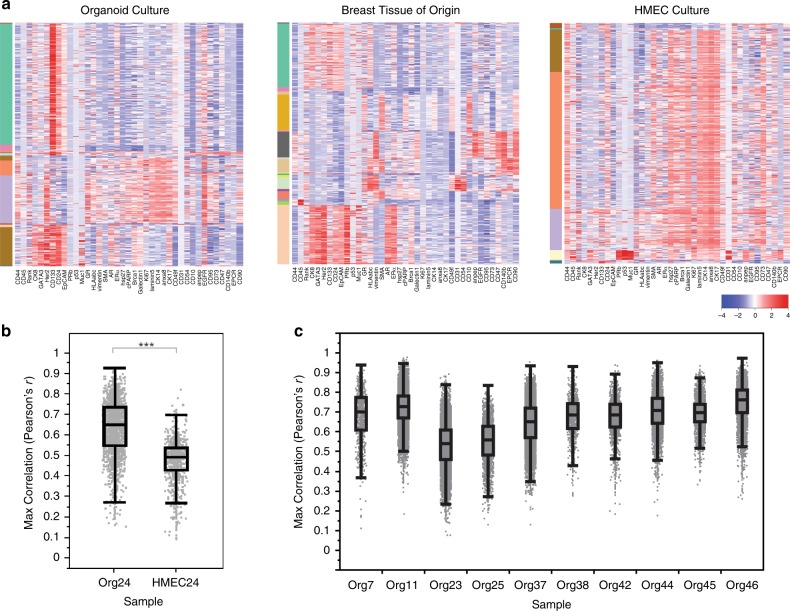
Analysis of matched organoid culture, HMECs, and primary tissue by CyTOF. Mammary tissue was dissociated and used to generate an organoid culture (ORG24) as well as a standard two-dimensional HMEC culture (HMEC24). Cells from the tissue was also directly fixed and frozen for future analysis. Cells from the cultures in conjunction with cells from the tissue were analyzed by CyTOF. **a** Heatmaps show single cells from the cultures or matched tissue as indicated, with color bar on left indicating different X-shift defined clusters. **b** Correlation between the protein expression profiles of HMEC or organoid cell and expression signatures derived from the major epithelial clusters in matched primary tissue. Box plots (center line, median; box limits, upper and lower quartiles; whiskers, 1.5× interquartile range) show the maximum *r* value of each cell to the major epithelial clusters, stratified by sample. Statistical significance was assessed by two-sided Mann–Whitney test (***p < 2.2e−16). **c** Correlation analysis as in **b**, performed for the indicated ten organoid cultures as compared with a set of four nonmatching primary breast tissues.

To ensure that differences between HMECs and tissues were not due to patient-to-patient variation, we generated an organoid culture and HMECs originating from the same breast tissue sample. Multiple mammary epithelial lineages could be detected in the organoid culture, but not in the HMECs (Fig. 5a). HMECs again diverged significantly from the primary tissues (Fig. 5b). A similar comparison of ten organoid cultures to a set of four nonmatching primary breast tissues demonstrated that the protein expression patterns of individual cells correlated between culture and nonmatching tissue with median Pearson’s *r* ranging from 0.54 to 0.76 (average 0.67, Fig. 5c). CyTOF analysis of three immortalized HMEC lines similarly exhibited significant differences in the expression of lineage markers^33^, as did MCF10A cells grown in three-dimensional culture, which are often used to model normal human mammary epithelium (Supplementary Fig. 8).

There were also notable differences between organoid cultures and the primary tissues in these analyses. In some of the organoid cultures, CD10 expression was lost (Supplementary Fig. 7a). This may represent the loss of stromal interacting cells or the innate tissue architecture, both of which cannot be fully recapitulated in vitro. Where CD10 expression was maintained, these cells were a subset of the CD90^+^ basal population (Supplementary Fig. 6). In addition, organoids exhibited high levels of expression of CD44 relative to primary breast tissues (Supplementary Figs. 6 and 7a). Increased CD44 has been reported previously in cultured mammary cells and was attributed to their increased rate of proliferation in culture^34^. Furthermore, CD44 is a Wnt target gene and may be induced by R-spondin 1 in the organoid culture medium^35^. Finally, as noted above, PR expression was generally low in the organoid cultures (Supplementary Fig. 7a). However, these changes were not as striking as the differences between the primary tissues and the HMEC cultures, where there was significant loss of lineage diversity and of the normal distinctions between the three major mammary populations.

Many of the changes observed in HMECs and MCF10As have been previously described based on a small number of markers (e.g., CK14, CK8, vimentin, and ERα), indicating cells with a mixed basal and luminal phenotype^3,36,37^; however, CyTOF analysis with a larger panel of markers provided a higher resolution analysis of these cells.

### Heterogeneity in organoids with and without *BRCA1* mutations

**Fig. 6:**
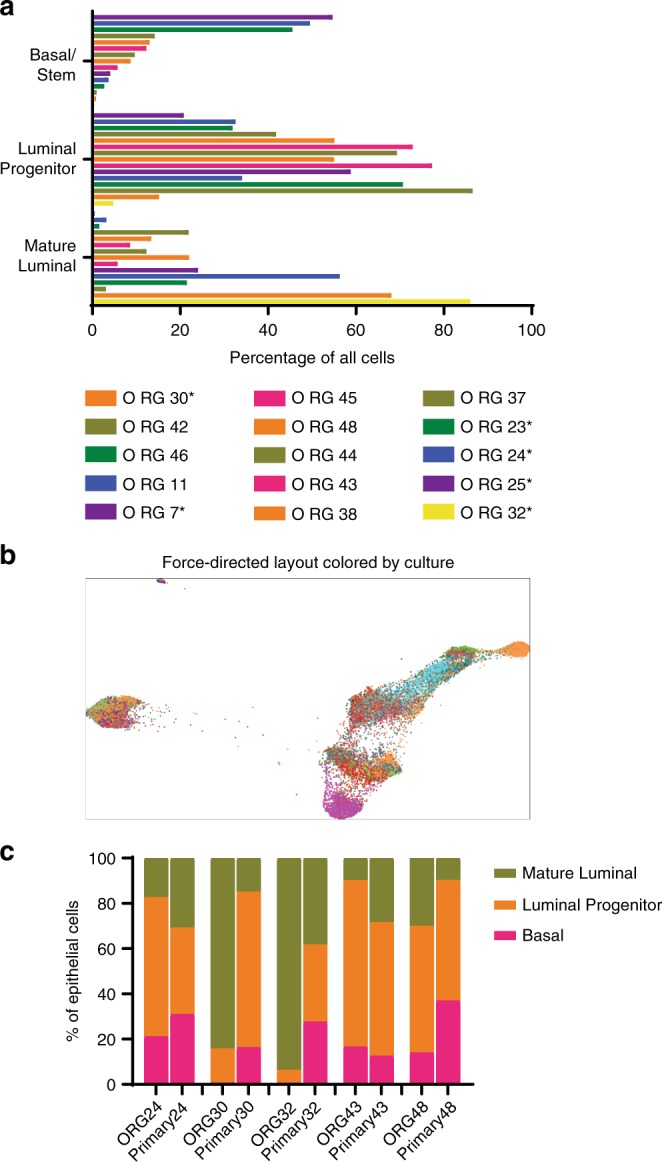
Heterogeneity is present in the distribution of mammary lineages in organoid cultures. **a** The proportion of cells in the basal, luminal progenitor, and mature luminal lineages were determined using EpCAM and CD49f levels, as measured by CyTOF for each of the indicated 15 mammary organoid cultures. Samples without a known mutation in a breast cancer predisposition gene are indicated with an asterisk. All cases were normal by histology. **b** Force-directed layout in which each dot represents one cell that is colored by culture ID (see Supplementary Fig. 9). **c** Percentage of epithelial cells in each of the indicated major mammary lineages is shown for the indicated organoid cultures and matching primary tissues.

Previous analyses of human mammary tissues have indicated a high degree of patient-to-patient variability in cell-type composition^38–40^. To assess whether similar findings are present in organoid cultures, we extracted EpCAM and CD49f expression levels from the CyTOF analyses of the 12 organoid cultures, as well as an additional three cultures run in an earlier pilot, to identify the proportion of cells present in each of the three major mammary lineages. We found that although the mammary lineages are maintained in organoid culture, the relative proportion of each lineage does vary from culture to culture (Fig. 6a). This is also notable in the X-shift-defined clusters and force-directed layout (Fig. 6b and Supplementary Fig. 9). To assess whether this variability reflects innate patient-to-patient variability we compared the lineage distribution of the five organoid cultures from Figs. 4 and 5 to their matching tissues, and found that the lineage distribution was similar in three cases but strikingly dissimilar in two (Fig. 6c). Differences in lineage distribution may be due to sampling artifact, as only a relatively small portion of the breast is used to make an organoid culture. In addition, differences in clinical variables such as age, parity, and inherited mutation status can contribute to variability and may result in specific cell populations becoming enriched in culture.

**Fig. 7:**
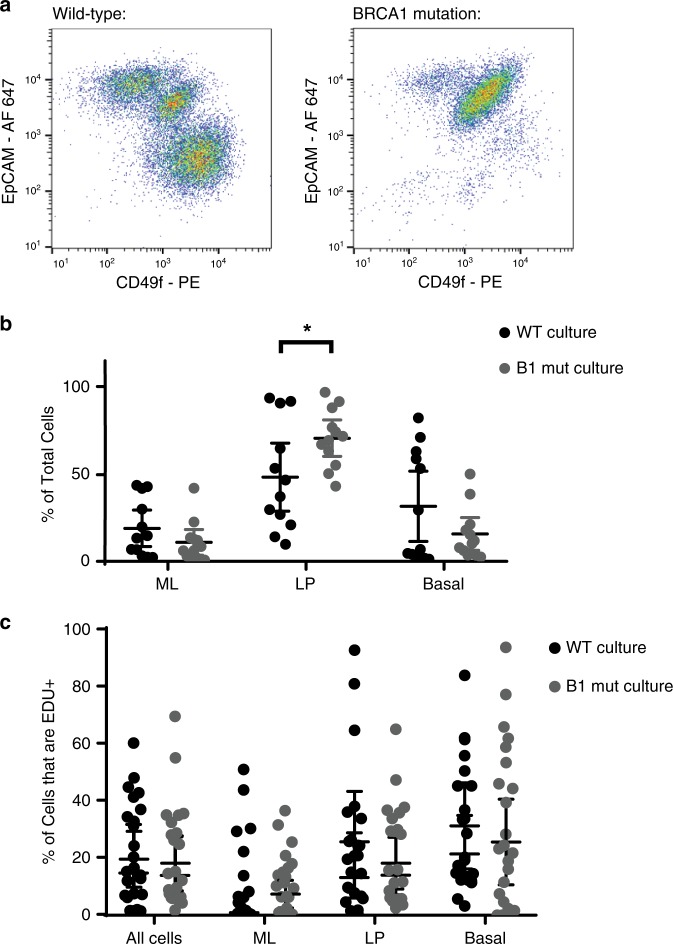
*BRCA1*-heterozygous mammary organoids have an increased proportion of luminal progenitors. **a** Representative organoid cultures without (wild-type), and with a known inherited mutation in *BRCA1* were digested to single cells, fixed, and stained for EpCAM and CD49f. Flow cytometry plots are shown. **b** Quantification of mature luminal (ML), luminal progenitor (LP), and basal/stem (Basal) populations as determined by flow cytometry assessment of EpCAM and CD49f levels are shown for 12 wild-type (WT) organoid cultures as compared with 12 cultures derived from the breast tissues of patients with an inherited mutation in *BRCA1* (B1 mut). **c** Proliferation in the mammary cell types was measured in all 24 cultures by incubating with EDU for 16 h and measuring EDU incorporation. In **b** and **c** mean with 95% confidence interval is shown. *p = 0.037, by Student’s *t* test, two-tailed.

It was previously reported that breast tissues from women with an inherited mutation in *BRCA1* have a higher proportion of luminal progenitor cells, which can be detected prior to any overt tumorigenesis^11^. We hypothesized that part of the apparent patient-to-patient variation in cell lineages in the organoid cultures could be due to a similar increase in the proportion of luminal progenitor cells in the cultures with a mutation in *BRCA1*. Analysis of 12 organoid cultures derived from reduction mammoplasties as compared with 12 organoid cultures derived from *BRCA1*-mutation carriers indeed showed that *BRCA1*-mutated organoid cultures have a higher proportion of luminal progenitors (Fig. 7a, b). Importantly, for this analysis, patients were matched with respect to the potential confounding variables of age and parity (Supplementary Fig. 10). Interestingly, this difference was not the result of an increase in proliferation of luminal progenitors from *BRCA1*-mutation carriers based on EDU incorporation (Fig. 7c), suggesting that the increase in luminal progenitors was not a result of preferential expansion during the culturing process but instead may reflect other inherent properties, or the high proportion of luminal progenitors in the native tissues, as previously reported^11^.

In addition to cultures with expanded luminal progenitor populations, two cultures with a significantly greater proportion of mature luminal populations were identified. Surprisingly, these “predominantly mature luminal cell” cultures could be passaged for >8 months in vitro despite being composed almost exclusively of more differentiated cells (Supplementary Fig. 11a). Cultures were composed of hollow acinar structures; over time larger structures with multilumen morphologies developed. These were also occasionally seen in other cultures at a lower frequency, have been previously described^41^, and resemble benign usual ductal hyperplasia (Supplementary Fig. 11b, c). Interestingly, proliferating mature luminal cells expressed higher levels of the γ2 subunit of laminin 332 (previous referred to as laminin 5 (V)), a basement membrane protein that is typically produced by basal myoepithelial cells (Supplementary Fig. 11d). In organoids, this may be a consequence of the proximity of luminal cells to the basement membrane extract in which they are grown.

Thus, the majority of breast tissues when cultured as organoids gave rise to mature luminal, luminal progenitor, as well as basal/stem epithelial cells. However, there is significant patient-to-patient heterogeneity. In some cancer-prone tissues, this reflected an increased proportion of luminal progenitor cells, whereas in other tissues, this was associated with different levels of other mammary epithelial subtypes.

### Organoid medium components affect the proportion of lineages

To determine the contribution of the individual growth factors and inhibitors to the representation of different mammary epithelial cell types in organoid culture, we generated a set of ten organoid media, with one factor missing in each of eight formulations (EGF, B27 supplement, the TGFβ inhibitor A83-01, R-spondin 1, FGF7 and FGF10, the p38 MAPK inhibitor SB202190, heregulin β1, and Noggin), and with two controls (with different sources of R-spondin 1). Using each of these ten media, we cultured mammary tissues from four different women. The cultures were passaged at least twice to generate sufficient cells, and analyzed by CyTOF.

**Fig. 8:**
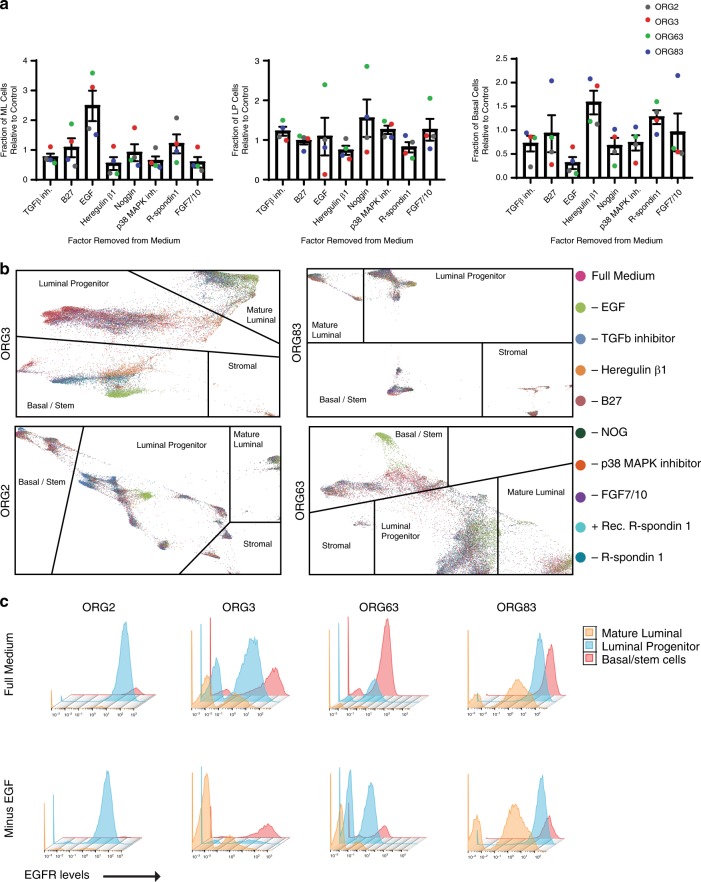
Distribution of mammary epithelial lineages upon removal of factors from organoid medium. Four human mammary tissues were dissociated, and a set of ten matching organoid cultures was generated using media formulations each with removal of the indicated medium component. **a** Fraction of mature luminal (ML), luminal progenitor (LP), and basal cells in organoid cultures grown in medium lacking the indicated factor, as normalized to control (full medium). Mean with standard error of the mean is shown (*n* = four cultures). **b** Force-directed layouts for the four sets of organoid cultures is shown where each dot, representing a single cell, is colored based on the specific formulation of media used. **c** EGFR expression levels in mature luminal, luminal progenitor, and basal/stem cells is shown by histogram, as measured by CyTOF, in matched organoid cultures grown in the presence or absence of EGF as indicated.

Analysis of the mammary lineages present in the set of ten organoid cultures revealed that removal of individual factors from the medium impacted the proportion of cells in each of the mammary lineages in the majority of cases (Fig. 8a and Supplementary Fig. 12a). This was also evident by force-directed layout, where cells grown under each of the ten conditions did not overlap, and exhibited differences in clustering both between and within the major mammary lineages (Fig. 8b).

The most striking changes resulted from removal of EGF, which caused an increase in the relative proportion of mature luminal cells, with a concomitant decrease in basal cells (Fig. 8a). Analysis of EGFR levels in the individual cells from the mammary populations revealed that, in the absence of EGF, the proportion of EGFR+ basal cells was decreased in all four cases tested, but EGFR+ luminal progenitor cells were only decreased in two of four cases (Fig. 8c). All of the mammary lineages exhibited consistent changes in protein expression under EGF-free conditions. Mature luminal cells exhibited decreased expression of the markers MUC1 and Galectin-1, luminal progenitor cells exhibited decreased expression of ANPEP (CD13), and basal cells exhibited increased expression of CD90 in all four cases (Supplementary Fig. 12b).

In contrast to EGF, removal of either heregulin *β*1, p38 MAPK inhibitor, or FGF7 and FGF10 resulted in a relative decrease in mature luminal cells with a concomitant increase in one of the other mammary lineages (Fig. 8a). Removal of TGFβ inhibitor and to a variable extent removal of Noggin from the medium resulted in a relative increase in luminal progenitor cells, whereas removal of R-spondin 1 resulted in a relative decrease in the luminal progenitor fraction (Fig. 8a). Interestingly, removal of B27 supplement did not consistently affect the distribution of mammary lineages in culture. However, removal of B27, or removal of EGF, did affect the organoid culture overall as evident by a decrease in the confluency and size of the organoids in all four cases (Supplementary Fig. 12c). There was also a decrease in the percentage of basal cells that are CD73^+^ CD90^−^ in three out of four cases upon removal of B27, and in all four cases upon removal of EGF (Supplementary Fig. 13). A distinct type of CD73^+^ CD90^−^ cell has been described as a minor population of mammary epithelial cells with bipotent progenitor activity^42^.

These findings demonstrate that the composition of the organoid medium can be altered to influence the relative proportion of mammary lineages present in organoid culture, and have implications for further investigations on mammary epithelial cell differentiation and autonomous as well as noncell autonomous signaling.

## Discussion

Organoid technology has enabled the in vitro culture of many types of epithelial tissues due to maintenance of basic epithelial architecture, stem cell hierarchy, and differentiation of specialized cell types. Sachs et al. developed organoid culture conditions optimized for long term in vitro propagation of breast tumor and normal epithelium^4^. In this study, we have characterized the properties of the mammary structures generated in these cultures, analyzed the composition of epithelial cell subtypes in the structures, and demonstrated that basal and progenitor cells can differentiate in culture to form the major mammary lineages. In addition, this study demonstrates the ability of CyTOF technology to provide a high resolution analysis of the extent to which organoid cultures faithfully preserve subtypes of epithelial cells from the normal mammary gland.

The organoid culture conditions used in this study allow the maintenance of three well-characterized human epithelial cell populations that are distinguished by EPCAM and CD49f, the canonical markers for defining human mammary epithelial subtypes. These cells have been designated as basal/stem cells, luminal progenitors, and mature luminal cells. The proportion of cells in these lineages varied from patient to patient, but within a single culture multiple lineages could be preserved with serial passaging. Furthermore, the cell lineages can be altered by removal of individual growth factors and signaling inhibitors in the organoid medium, demonstrating the utility of this system for dissecting regulatory mechanisms of lineage differentiation. In addition, a previously published difference in the mammary tissue of individuals with or without mutations in *BRCA1*, namely an increase in the luminal progenitor population that is implicated in BRCA1-associated BC development^11,28,43^, was maintained in patient-derived organoid cultures. Thus, human mammary epithelial organoid cultures could be used to model tumorigenesis for cell-of-origin studies of BRCA1-associated BCs, and now make it feasible to directly examine the impact of cancer-associated alterations on luminal progenitor cells.

Changes in normal mammary populations were not restricted to *BRCA1*-mutation-associated cases. Although we do not have a sufficient number of cases for further analysis at this time, X-shift clustering algorithms did reveal a unique cluster of luminal progenitor cells present in an organoid culture derived from a patient with an inherited mutation in *TP53* (ORG42 in Supplementary Fig. 9). The native breast tissue from this case exhibited normal histology, but the organoids in culture were predominantly composed of luminal progenitor cells. Interestingly, the luminal progenitor cells had abnormal characteristics, such as high expression of ERα, distinct from the luminal progenitor subtype identified at an increased proportion in *BRCA1*-mutation-associated cases. This example highlights the potential utility of organoid cultures for identifying candidate premalignant cell types in otherwise ostensibly normal human tissues associated with mutations in different cancer predisposition genes.

Use of mass cytometry (CyTOF) provided a much better assessment of the retention of mammary-specific protein expression patterns in organoids as compared with standard flow cytometry. The library of antibodies in our CyTOF panel included multiple markers of each of the epithelial subpopulations, thus providing a higher resolution comparison of organoid cultures to primary mammary epithelial cells. This analysis showed that there was a remarkable degree of similarity in marker expression between the organoids and primary mammary tissues. This similarity contrasts with the fate of primary cells cultured under standard monolayer conditions, in which the diverse lineages (especially ER+ luminal cells) are lost, while the remaining cells lose their cell-type restricted markers and acquire a homogeneous and aberrant mixed luminal-basal state.

Although the similarities were striking, there were also notable differences between epithelial cells in organoid cultures and primary tissues. This is not surprising, since the cells are taken out of their native microenvironment and are no longer in contact with stromal cells. In addition, dissociation and digestion protocols are different between cultures and tissues by necessity, which might affect antigen detection.

Importantly, sorting experiments showed that both basal and luminal progenitor cell types were capable of generating all three cell types. Patient-to-patient heterogeneity in the ability of sorted luminal progenitor cells to differentiate in culture was an unexpected finding and warrants further investigation of the underlying mechanisms in future studies. Extended culture of mature luminal and luminal progenitor cells has been challenging to date, but is highly efficient using this organoid culture system. The ability to expand multiple mammary epithelial cell types with high efficiency represents a useful advance for the field, enabling future studies of normal mammary differentiation, as well as its perturbation and the transformation of cells in different lineages to their premalignant and malignant counterparts.

Other three-dimensional mammary culture systems have been developed and utilized for studying normal mammary epithelial cells. Media that have been used for 3D culture of immortalized HMECs originally generated in monolayer cultures allow formation of uniform single-layer cyst-like structures, or structures with squamous centers; however, the media used in those cultures do not preserve ER+ luminal cells long term and the HMECs do not resemble primary epithelial cells^2,24,44,45^. Several culture models have been reported to facilitate the generation of acinar and ductal structures containing basal and luminal cells that respond to estrogen and undergo branching morphogenesis^22,46–49^; however, those conditions do not allow primary cells to be propagated continuously. Mouse mammary organoids have also been described with distinct organoid structure types from those we have identified in human organoid cultures, and the luminal and basal mouse mammary epithelial cell types exhibited differences in their differentiation in organoid culture^50^. We found that human organoid cultures are notable for the presence of multiple structure types within a single patient-derived culture, with structural heterogeneity linked to the diversity of normal mammary cell types, and by the ability to culture these cell types long term.

In summary, our study demonstrates that normal human mammary organoids can be generated with high efficiency and maintain the major subpopulations of epithelial cells over long-term culture. Mammary organoid cultures represent a versatile and powerful tool for further study of normal mammary lineages, such as luminal progenitor cells which have been proposed to be the cancer cell of origin for some BCs^51^. The findings described herein provide a framework for the elucidation of normal differentiation states as well as premalignant phenotypes in the human mammary gland.

## Methods

### Antibodies and chemicals

The following primary antibodies were used: rabbit polyclonal anti-cleaved caspase 3 (Cell Signaling, Cat. No. 9661L), rabbit monoclonal anti-CK8 (Alexa Fluor 488) (Abcam, Cat. No. ab192467), mouse monoclonal anti-CK8 (Santa Cruz, sc-8020), rabbit monoclonal anti-CK14 (Alexa Fluor 647) (Abcam, Cat. No. ab206100), rabbit polyclonal anti-CK14 (BioLegend, Cat. No. 905301), rabbit monoclonal anti-cytokeratin 19 (Abcam, ab52625), rabbit monoclonal anti-Estrogen Receptor α (Abcam, Cat. No. ab16660), mouse monoclonal anti-EpCAM (Alexa Fluor 647) (Biolegend, Cat. No. 324212), rat monoclonal anti-CD49f (phycoerythrin) (Biolegend, Cat. No. 313611), rabbit monoclonal anti-ERBB3 (Abcam, ab236220), and rabbit polyclonal anti-Ki67 (Abcam, Cat. No. ab15580). The antibodies used in our CyTOF panel are listed in Supplemental Table 4. Validation of antibodies was performed as described in the reporting summary available as a Supplementary Information file. In addition, we used the following: 4′,6-Diamidino-2-phenylindole dihydrochloride (DAPI) (Sigma, Cat. No. D9542), Molecular Probes Phalloidin (F-actin probe) (Invitrogen, Cat. No. A22287), and β-estradiol (Sigma, Cat. No. E4389).

### Isolation of organoids from breast samples

Breast tissues were obtained from reduction mammoplasty or prophylactic mastectomy samples at Brigham & Women’s Hospital or Faulkner Hospital and were processed on the day of surgery. This study was reviewed by the Harvard Medical School Institutional Review Board and deemed not human subjects research, and patients gave their informed consent to have tissue used for scientific research purposes. Tissue obtained was minced, and then placed into a 50 ml conical tube containing 20 ml AdDF+++ (Advanced DMEM/F12 containing 1× Glutamax, 10 mM HEPES, and antibiotics) and 1 mg/ml collagenase (Sigma, C9407). In some cases, viable tissue was also frozen in 90% FBS and 10% DMSO for subsequent generation of organoid cultures or HMECs. Tubes containing minced tissue and collagenase were wrapped in parafilm and placed on their side in an orbital shaker at 37 °C for 2 h. After digestion, 30 ml AdDF+++ and 2% FBS were added and the organoids were pelleted. Further mechanical shearing was achieved by adding 10 ml AdDF+++ and sequentially pipetting with 10, 5, and 1 ml pipette tips before the final pellet of primary breast organoids was obtained.

### Organoid culture

Organoids were cultured as previously described^4^. Primary breast organoids were resuspended in 10 mg/ml Cultrex growth factor-reduced BME type 2 (Trevigen, Cat. No. 3533-010-02). A 50 μl drop of this suspension was placed in the center of a well in a 24-well suspension culture plate (Greiner, Cat. No. M9312) and allowed to harden for 20 min at 37 °C. 500 μl of BC organoid culture medium^4^ was then placed over the droplet. For some experiments, BC organoid culture medium was used that was missing one or two components as indicated in the text. Medium was changed every 3–4 days, and organoids were passaged using TrypLE Express (Invitrogen, 12605036) approximately every 2–4 weeks. Y-27632 was removed from BC organoid culture medium starting from 3 days after initial seeding. Organoids were viably frozen using Recovery Cell Culture Freezing Medium (Life Technologies, Cat. No. 12648-010). Minced tissue was viably frozen in FBS containing 10% DMSO for later generation of organoid cultures. For hormone stimulation, organoids were treated with 0.5 ng/ml beta-estradiol in BC organoid medium for 7 days. Viability was measured by luminescence after lysis with CellTiter-Glo (Promega, Cat. No. G7572). Where indicated, karyotype was determined by the Brigham & Women’s Hospital CytoGenomics Core Facility, and karyotype images were taken at 1000x magnification. A list of the organoid cultures generated is provided in Supplementary Data. Organoid cultures will be made available upon request.

### Flow cytometry

Organoid cultures were treated with 5-ethynyl-2′-deoxyuridine (EdU) at 10 µM for 16 h, and digested to single cells with TrypLE. Cells were fixed and labeled using the Click-iT EdU Alexa Fluor 488 Flow Cytometry Assay Kit (ThermoFisher, Cat. No. C10420), and with Alexa Fluor 647-conjugated anti-EpCAM (1:50) and phycoerythrin-conjugated anti-CD49f (1:50), according to the manufacturer’s instructions. ALDH activity was assessed using the ALDEFLUOR kit (StemCell Technologies, Cat. No. 01700) according to the manufacturer’s instructions.

### FACS isolation of mammary epithelial cell types

Single cells were isolated from organoid cultures with TrypLE and labeled for 30 min at room temperature with Alexa Fluor 647-conjugated anti-EpCAM (1:50) and phycoerythrin-conjugated anti-CD49f (1:50) in AdDF+++ medium containing 10% goat serum. EpCAM^+^ CD49f^+^ (luminal progenitor), EpCAM^−^ CD49f^+^ (basal/stem cell), and EpCAM^+^ CD49f^−^ (mature luminal) cells were sorted by FACS and cultured at equal densities in BC organoid medium as described above. Organoid-forming efficiency was calculated by manually counting with a hemocytometer the number of organoids established from an equal number of cells of each type.

### Immunohistochemistry

Basement membrane extract gels containing embedded organoids were washed with PBS, and detached gently from the bottom of the well by running a pipette tip around the edge of the gel. Up to three intact droplets were placed into a 10 × 10 × 5 mm Cryo Mold (Electron Microscopy Sciences, Cat. No. 62534-10), and ~200 µl of HistoGel (VWR, Cat. No. 83009-992) was added to the Cryo Mold. The Cyro Mold was placed on ice for ~10 min, and then the contents were removed and fixed in 10% neutral buffered formalin overnight. Paraffin embedding, sectioning, and Hematoxylin & Eosin staining was performed by the Brigham & Women’s Hospital Pathology Core facility. Immunohistochemistry and immunofluorescence (either on formalin-fixed paraffin-embedded sections or on organoids grown in chamber slides) and confocal microscopy was performed as previously described^52–54^. For immunohistochemistry, antigen retrieval was performed on deparaffinized slides using a steamer and citrate pH6 (Dako, Cat. No. S3020) for 40 min, peroxidase was blocked using 3% hydrogen peroxide (Sigma, Cat. No. H1009), blocking was performed in 5% goat serum (Invitrogen, Cat. No. 16210) for 1 h at room temperature, and slides were incubated in primary antibody in Antibody Diluent (Dako, Cat. No. S0809) followed by secondary antibody for 30 min at room temperature (Dako, Cat. No. K4000 or K4002). Slides were then stained with DAB (Sigma, Cat. No. D4418) and Hematoxylin (Sigma, Cat. No. MHS32) per the manufacturer’s instructions, and mounted with Permount (Fisher, Cat. No. SP15). Immunofluorescence was performed on organoids grown on chamber slides by fixing them in 4% paraformaldehyde for 20 min at room temperature, permeabilizing with 0.5% TritonX-100 for 10 min at 4 °C, incubating in primary antibodies overnight at 4 °C and then secondary antibodies for 45–60 min at room temperature in 10% goat serum, and then incubating with DAPI (1 µg/ml) for 20 min at room temperature. For quantification of CK8+, CK14+, and ERα+ organoids, >50 organoids were counted per culture and per condition. For quantification of ERα+ cells, >200 cells were counted per culture and per condition.

### Mass cytometry

For CyTOF analyses, organoids were first digested to single cells using TrypLE. Mammary tissues were minced, and then dissociated to single cells by incubating minced tissue with collagenase (1 mg/ml) for ~2.5 h on an orbital shaker at 37 °C until all pieces were visibly digested, followed by dispase (5 U/ml, Stem cell technologies, Cat. No. 07913) and trypsin 0.25% (Corning, Cat. No. 25-053-Cl) for 5 min at 37 °C. Cell pellets were then treated with RBC lysis buffer (Biolegend, #420301). The resulting digested suspension was filtered through a large strainer followed by a 40 µm filter. All cells used in CyTOF analyses were then resuspended in PBS and treated with 5 µM cisplatin (Fluidigm, Cat. No. 201064) for 5 min to label DNA, and then quenched with Cell Staining Medium (CSM, Fluidigm). Cells were fixed with 1.6% paraformaldehyde for 10 min. Individual samples were barcoded using the Cell-ID 20-Plex Pd Barcoding Kit (Fluidigm), and then pooled and treated with DNase I (Stemcell, Cat. No. 07900) for 30 min. The pooled sample was then stained with the extracellular mammary-specific CyTOF antibody panel (see Supplementary Table 4), washed with PBS twice, resuspended in 90% cold methanol for 30 min. on ice, washed twice with CSM, stained with the intracellular CyTOF antibodies (see Supplementary Table 4), and washed two times with CSM. The sample was then fixed with 4% PFA for 30 min and stained with Iridium intercalator (Fluidigm, Cat. No. 201192) overnight to stain DNA, thereby allowing exclusion of cell clumps and debris in downstream analyses. After incubation, cells were washed with distilled water three times, EQ four element calibration beads (Fluidigm, Cat. No. 201078) were added to the sample, and the sample was run on the Helios CyTOF instrument with the assistance of the Dana-Farber Cancer Institute Flow Cytometry Core. After acquisition, the data were normalized and debarcoded into fcs files containing each of the samples individually. The files were then imported into the FlowJo software (Ashland, OR), where cell debris, cell clumps, dead cells, and calibration bead-cell clumps were excluded. The resultant files were then clustered using the X-shift algorithm in the Vortex software package developed by the lab of Garry Nolan, and force-directed layout graphs were generated^27^. Heatmaps were generated in R 3.5.1.

### Statistical analysis

Values are reported as mean ± standard deviation, ±standard error of the mean, or ±95% confidence interval as indicated in the text. Statistical analyses were performed in Prism (GraphPad) or FlowJo. Experiments involving two groups were analyzed using Student’s two-tailed *t* test. *P* values > 0.05 were considered significant. For comparison between cultures and matched tissues, major epithelial clusters in each CyTOF experiment were identified as clusters with >30 cells in primary tissues that express epithelial markers and lack expression of stromal markers. Expression signatures for each of the major epithelial clusters were calculated as the median value for each CyTOF marker within each cluster from each primary tissue sample. In cases where the comparison was to nonmatching tissues, a set of four tissues was used (Primary30, Primary32, Primary43, and Primary48). To assess the level of correlation of each HMEC and organoid cell to the primary tissue epithelial signatures, Pearson’s *r* was calculated in R 3.5.1. Box plots show the maximum *r* value of each cell to the major epithelial clusters, stratified by sample. Statistical significance was assessed by Mann–Whitney test in JMP Pro 14.0.0.

### Reporting summary

Further information on research design is available in the Nature Research Reporting Summary linked to this article.

## Supplementary information

Supplementary Information

Description of Additional Supplementary Files

Supplementary Data 1

Reporting Summary

## Data Availability

The raw CyTOF data have been deposited in the FlowRepository database under the accession codes FR-FCM-Z2HY, FR-FCM-Z2H2, and FR-FCM-Z2H5. All the other data supporting the findings of this study are available within the article and its Supplementary Information files and from the corresponding author upon reasonable request. A reporting summary for this article is available as a Supplementary Information file.

## Code availability

Code for generation of heatmaps and for correlation analyses is available for download at https://github.com/lselfors/Rosenbluth_organoid_cultures_NatureComm2020.
