# Supplementary material for: Organoid cultures from normal and cancer-prone human breast tissues preserve complex epithelial lineages
Source: Nat Commun. 2020 Apr 6;11:1711. doi: 10.1038/s41467-020-15548-7 (PMC7136203; doi:10.1038/s41467-020-15548-7)
Supplement: Supplementary file 1 — Supplementary Information [file 41467_2020_15548_MOESM1_ESM.pdf]

Supplementary Information

Organoid Cultures from Normal and Cancer-Prone Human Breast Tissues Preserve Complex Epithelial Lineages

Rosenbluth et al.

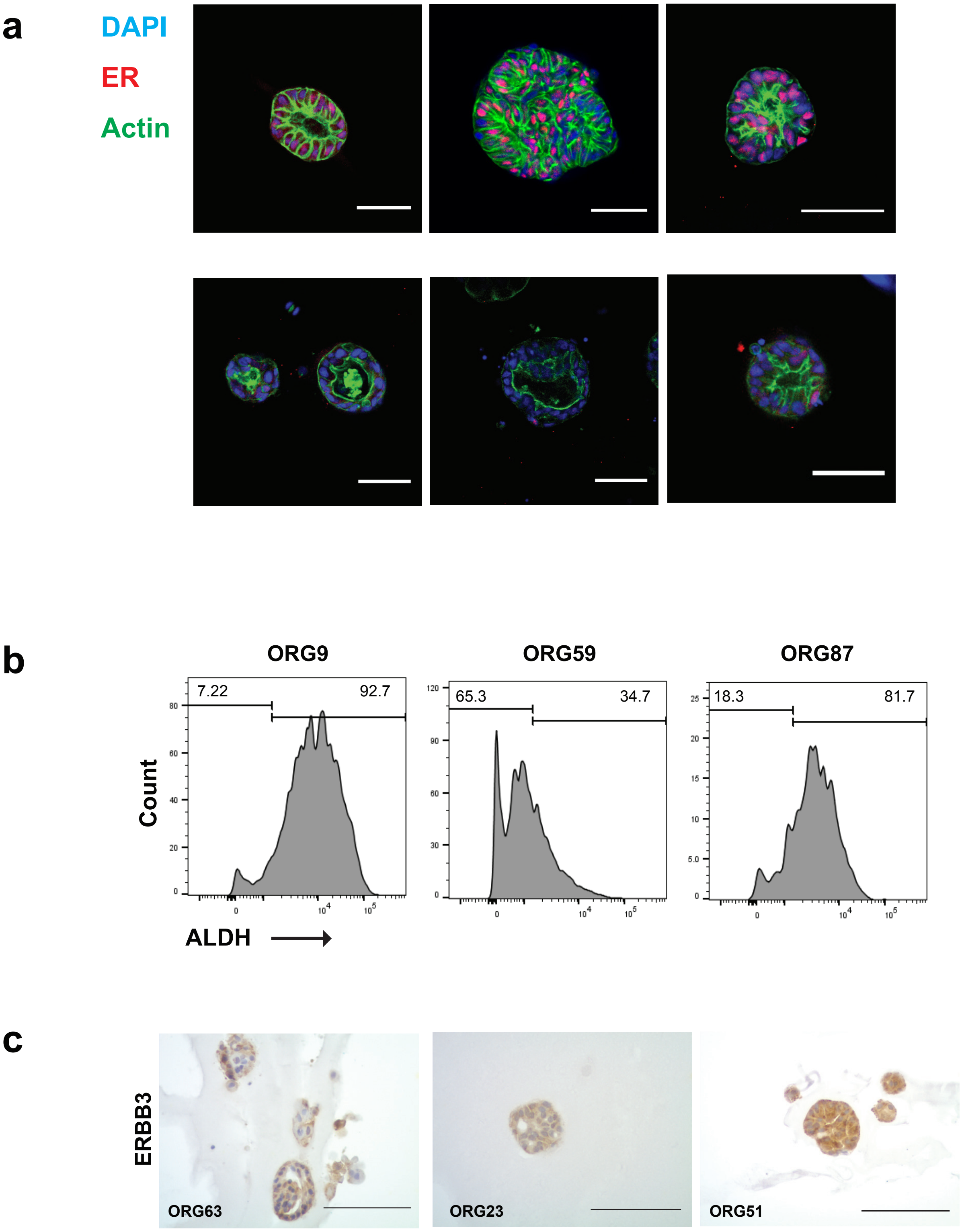

Supplementary Figure 1. Examples of marker expression in mammary organoids. **a** Examples of high ER expression (top panels) and low ER expression (bottom panels) as assessed by immuno-fluorescence and confocal microscopy for the indicated markers, scale bar = 100 μm. **b** Flow cytometry analysis of ALDH activity in the luminal progenitor cells (EpCAM$^+$ CD49f$^+$) from three organoid cultures. ALDH activity is compared to a negative control using a specific inhibitor of ALDH. The percentage of ALDH$^+$ and ALDH$^-$ luminal progenitor cells is shown for each culture. **c** Immunohistochemistry for ERBB3 in luminal progenitor-type organoids in the indicated cultures, counterstained with Hematoxylin, scale bar = 100 μm.

Epithelial

EPCAM
CD24
MUC1
ANPEP
CD133
Laminin5
GATA3
Anxa8
CK8
HER2
CD49f
CK14
CK17

Mesenchymal

EGFR
CD44
vimentin
CD90
SMA
Galectin1
CD10
EPCR

Hormone Receptors

Estrogen Receptor alpha
Androgen Receptor
Glucocorticoid Receptor
Progesterone Receptor beta

Stroma

CD31
CD140b
CD45

Other

Ki67
p53
CD54
CD73
CD95
CD47
H3K27Me3
RANK
BRCA1
HLAabc
HSP27
TSPAN8

Supplementary Figure 2. CyTOF antibody panel for analysis of human mammary lineages. Antibodies recognizing the proteins listed in each of the 5 categories (see Supplementary Table 4) were validated, conjugated to heavy metals, and used for CyTOF profiling of human mammary cells.

a

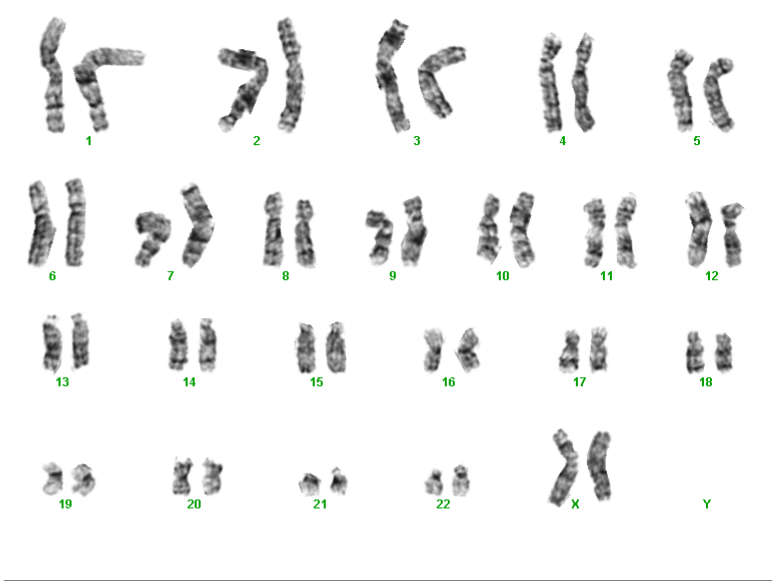

b

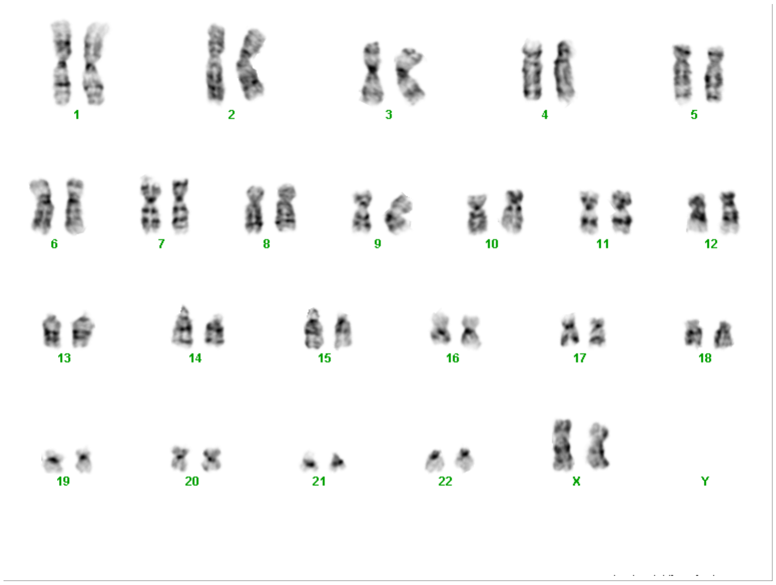

Supplementary Figure 3. Karyotype analysis of two cultures used in the CyTOF analysis. Examples of metaphase spreads for two organoid cultures, ORG37 and ORG38, demonstrating normal diploid karyotypes.

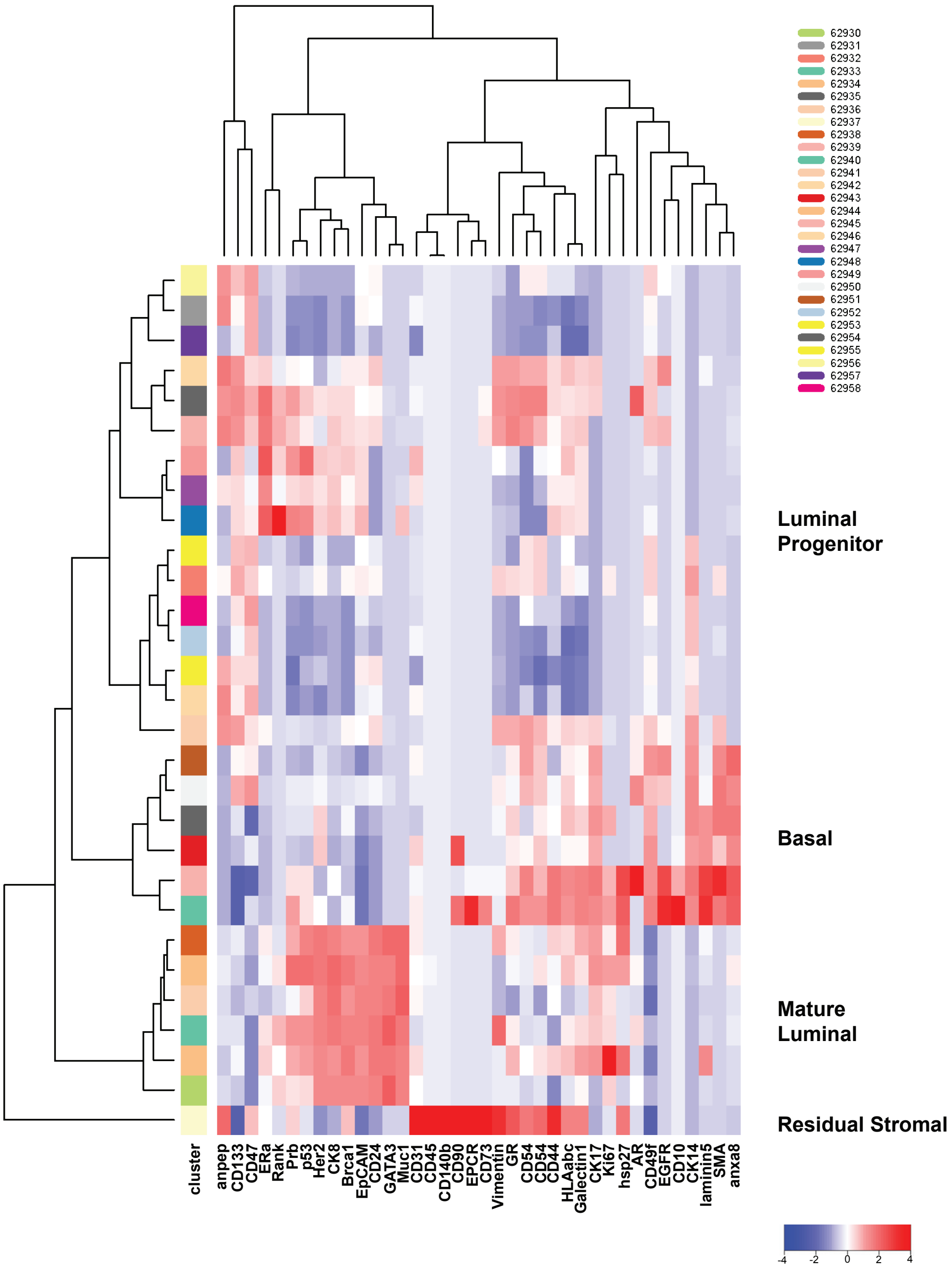

Supplementary Figure 4. Heat map of X-shift-defined clusters from 12 organoid cultures analyzed by CyTOF. CyTOF analysis was performed on 12 organoid cultures derived from normal human mammary tissues. Clustering of cells was performed using X-shift to define 29 distinct clusters (y-axis). The heat map displays the abundance of each of the markers in the panel (x-axis) (red = higher expression level, blue = lower expression level).

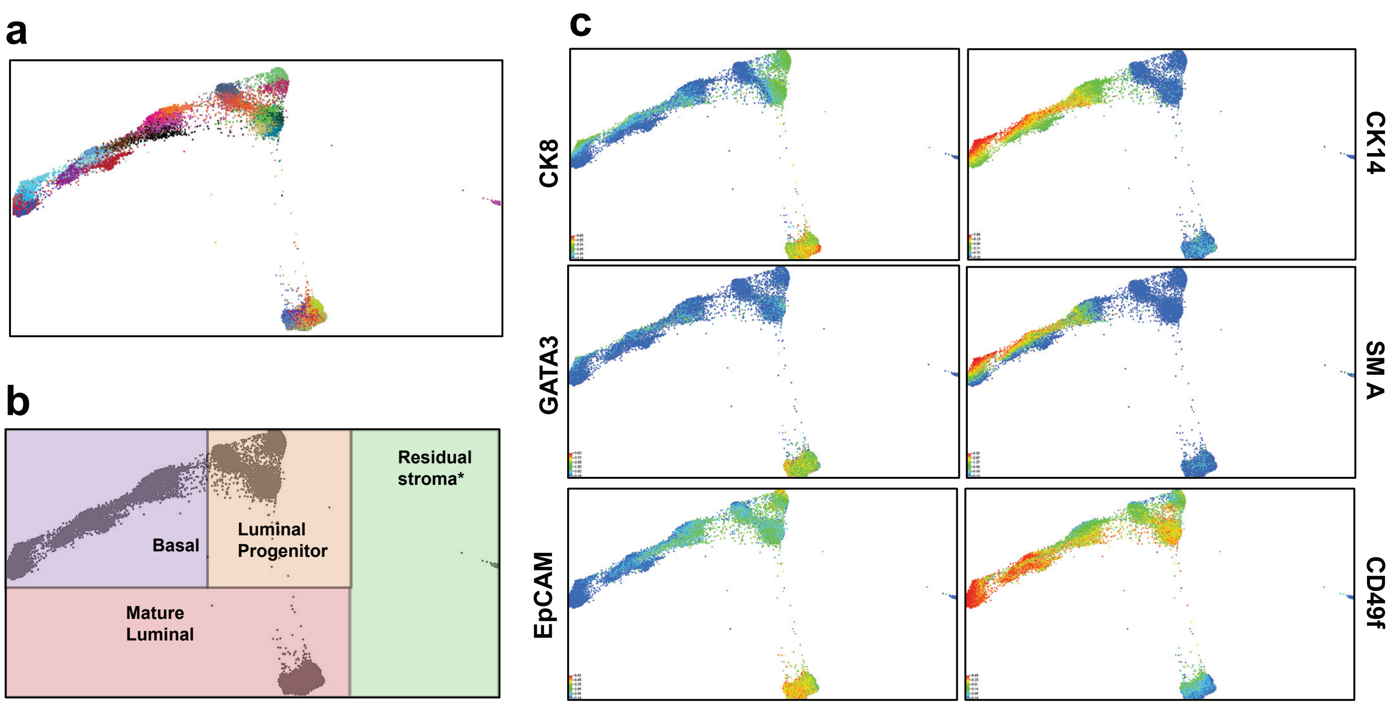

Supplementary Figure 5. Re-analysis of CyTOF data after exclusion of BRCA1 and p53 from the CyTOF panel. CyTOF data were re-analyzed as in Figure 3, but excluding the markers BRCA1 and p53 from the X-shift clustering. Global clustering pattern nearly identical to Figure 3 was obtained, as shown by force-directed layout in **a**-**c**. Note that the minor re-orientation of the clusters relative to Figure 3 is intrinsic to each X-shift re-run and does not reflect changes in the global clustering pattern. **a** Cells are colored by X-shift-defined cluster. **b** Cell types as determined by expression of markers such as those shown in **c**. **c** The levels of the indicated markers are shown, with warmer colors demonstrating higher protein expression levels.

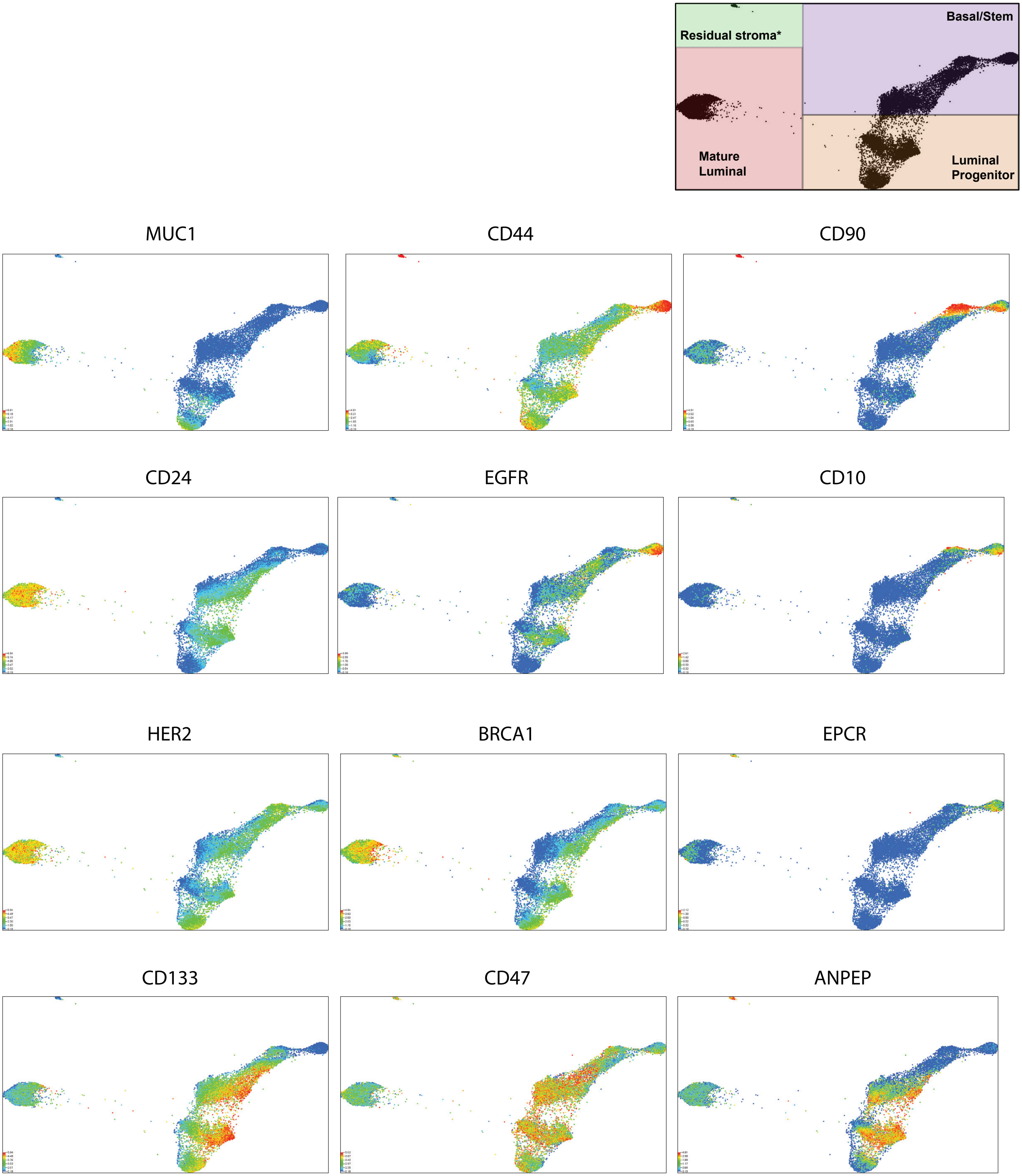

Supplementary Figure 6. Expression levels of select markers of interest from CyTOF analysis of 12 organoid cultures. Force-directed layouts of the 12 organoid cultures analyzed by CyTOF in Figure 3. In each panel, each dot represents one cell, and colors represent the expression levels of the indicated markers (warmer color = higher expression level).

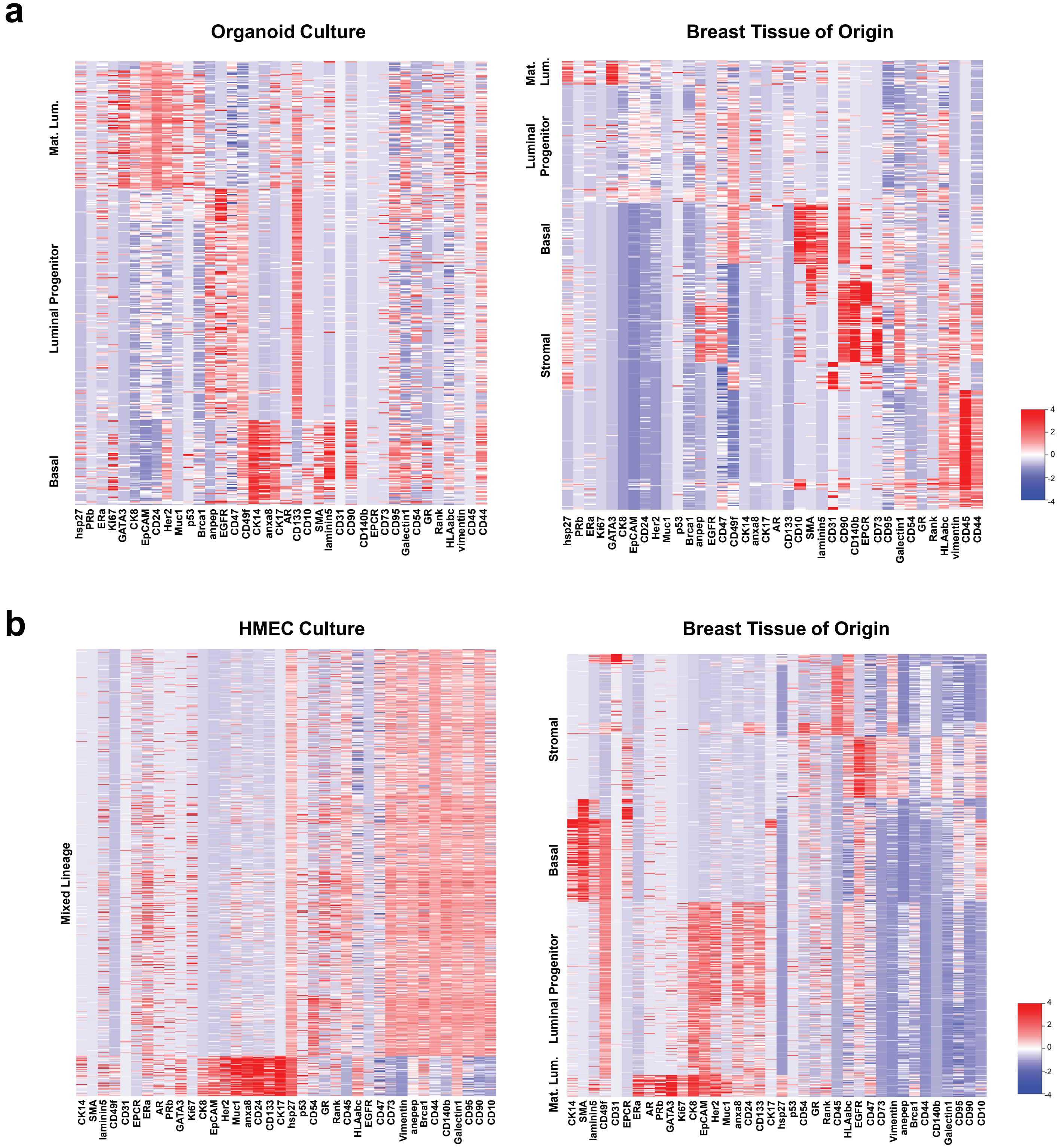

Supplementary Figure 7. CyTOF analysis of cultures and matched tissues of origin. Heat maps show individual cells along the y-axis, ordered by X-shift clustering. Identified cell types are labelled. Markers in the CyTOF panel are on the x-axis. **a** Single-cell heat map of a representative tissue (right) and its matched organoid culture (left). **b** Single-cell heat map of representative tissue (right) and its matched HMEC culture (left).

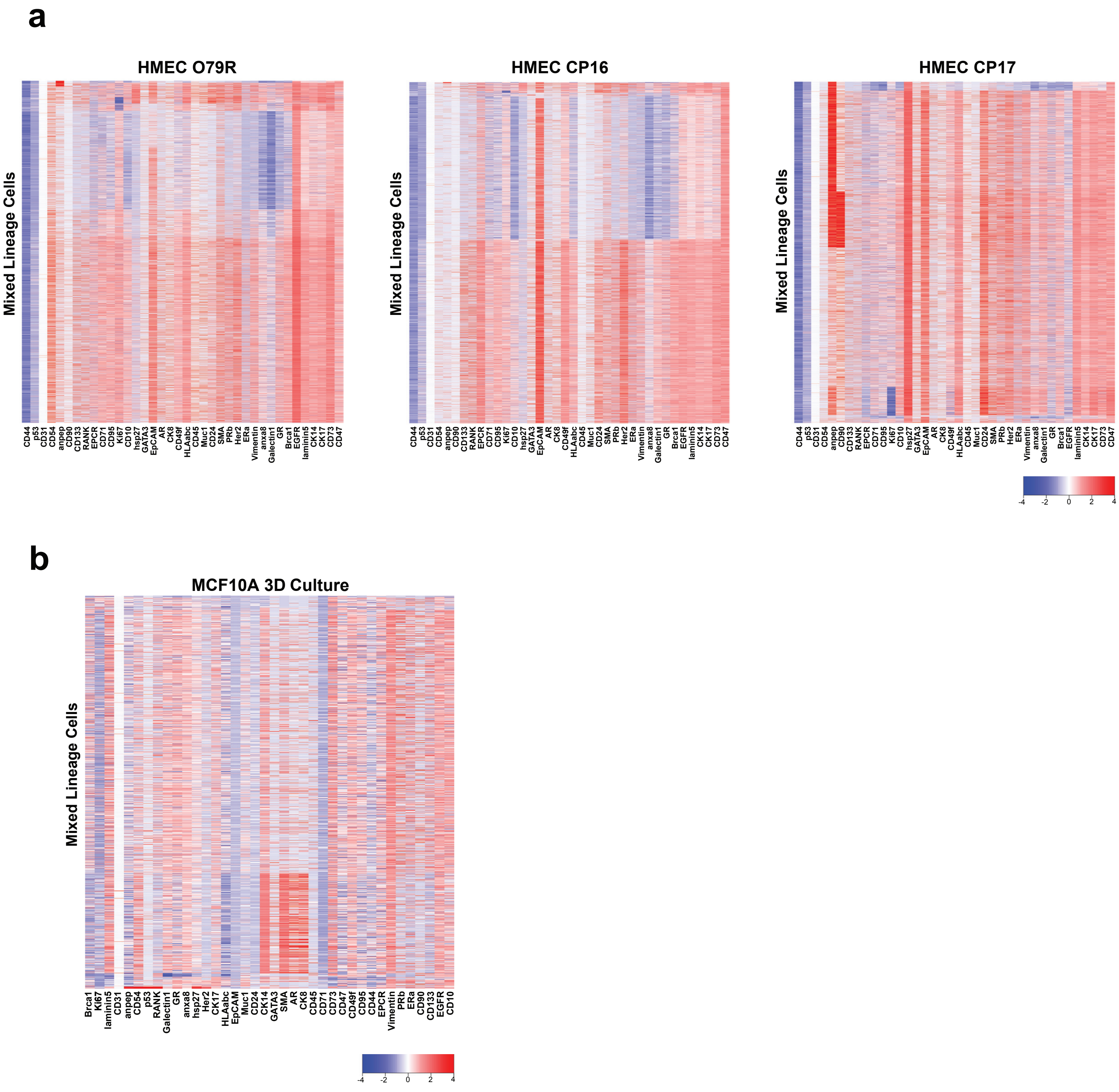

Supplementary Figure 8. CyTOF analysis of immortalized mammary cell lines. **a** Single-cell heat maps showing three immortalized HMEC cell lines grown in standard two-dimensional culture. **b** MCF10A cells were grown in matrigel to form acinar structures. Acini were dissociated to single cells and analyzed by CyTOF. A single-cell heat map is depicted.

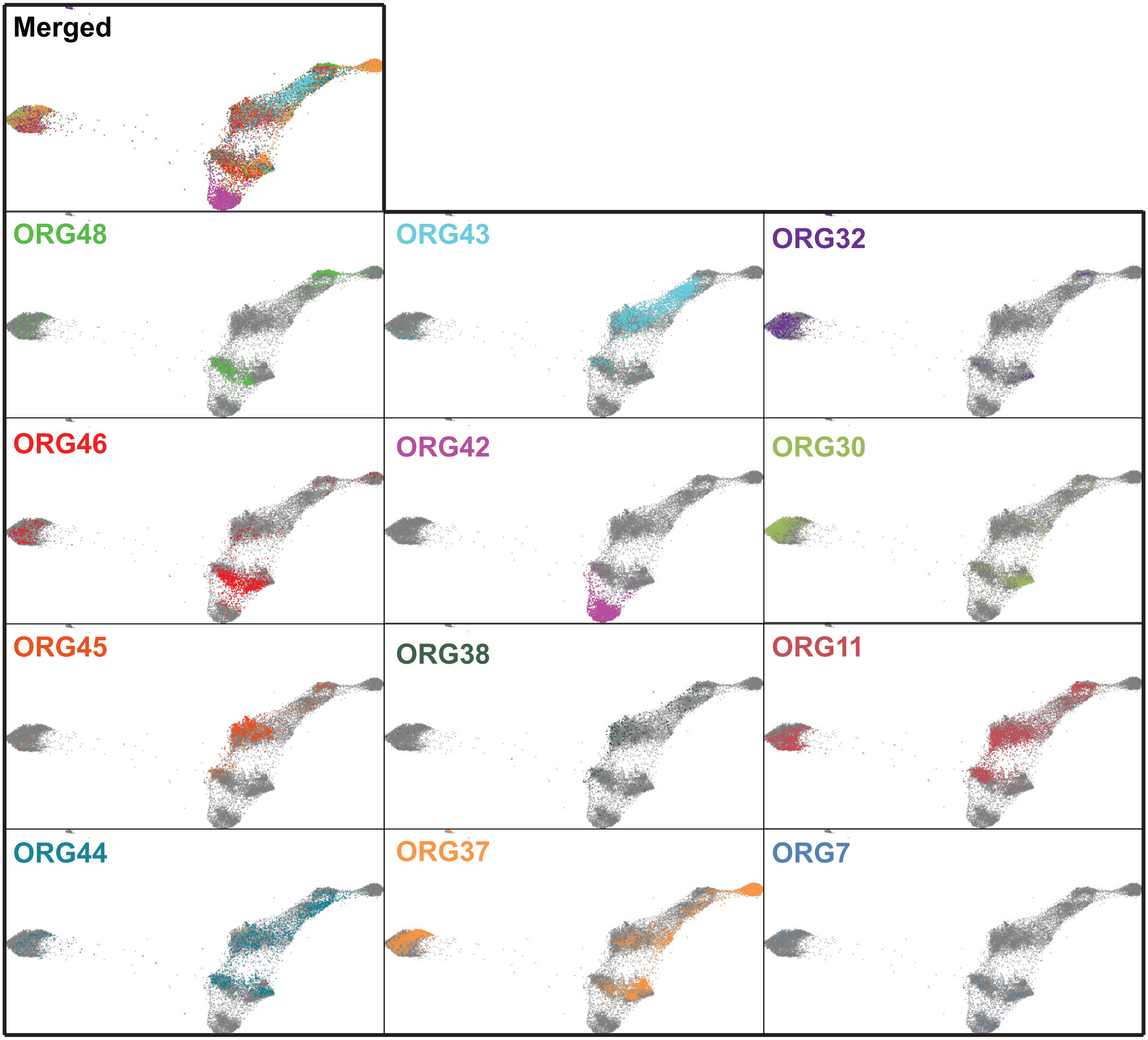

Supplementary Figure 9. Patient-to-patient heterogeneity in organoid cultures. Force-directed layouts of the 12 organoid cultures analyzed by CyTOF in Figure 3. Each dot represents one cell, and in each panel the cells from the indicated culture are colored.

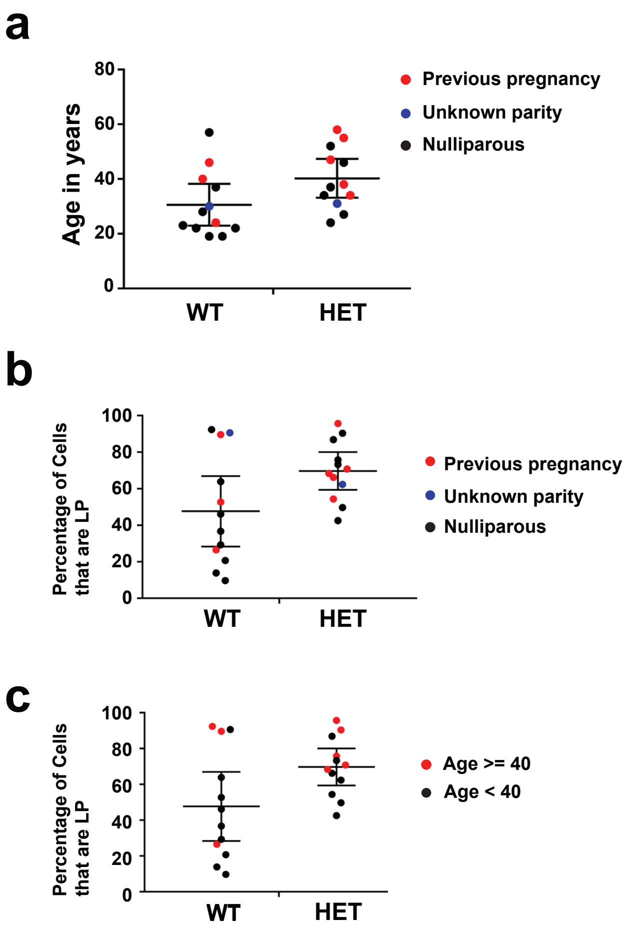

Supplementary Figure 10. Assessment of parity and age in set of wild-type and BRCA1-mutated organoid cultures. **a** Ages of patient donors of the organoid cultures shown in Figure 7. Patients with (HET) or without (WT) a known mutation in *BRCA1* are indicated, and previous pregnancy status is indicated by color. **b** Overlay of parity on the % of cells that are Luminal Progenitor (LP), extracted from Figure 7b. **c** Overlay of age on the % of cells that are LP, extracted from Figure 7b. Mean with 95% confidence interval is shown. For each genotype, n = 12 biologically independent organoid cultures were assessed over the course of 12 independent experiments.

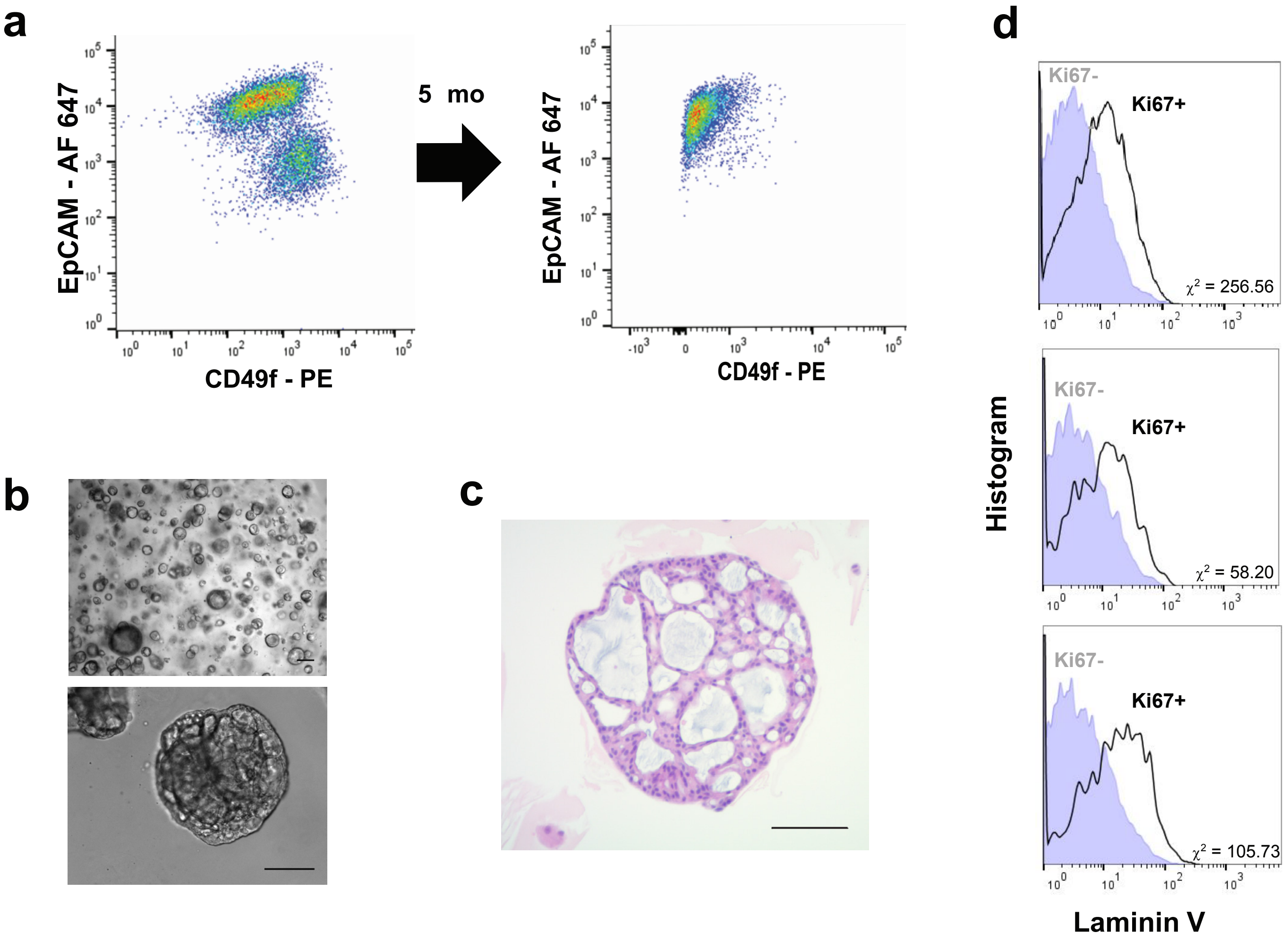

Supplementary Figure 11. Mature luminal cell-predominant organoid cultures. **a** Cells from an organoid culture, ORG32, were stained with EpCAM and CD49f and analyzed by flow cytometry. Analysis was repeated after five additional months in culture. **b** Bright field microscopy images of mature luminal cell-predominant culture indicating a high proportion of acinar and multi-lumen structures (representative of n = 2). Scale bar = 100 μm. **c** H & E stained section of a multi-lumen organoid structure exhibiting similarity to Usual Ductal Hyperplasia (representative of n = 2). Scale bar = 100 μm. **d** Laminin-V levels of $Ki67^-$ versus $Ki67^+$ cells, as measured by CyTOF, are shown by histogram for three organoid cultures, with χ^2 values shown.

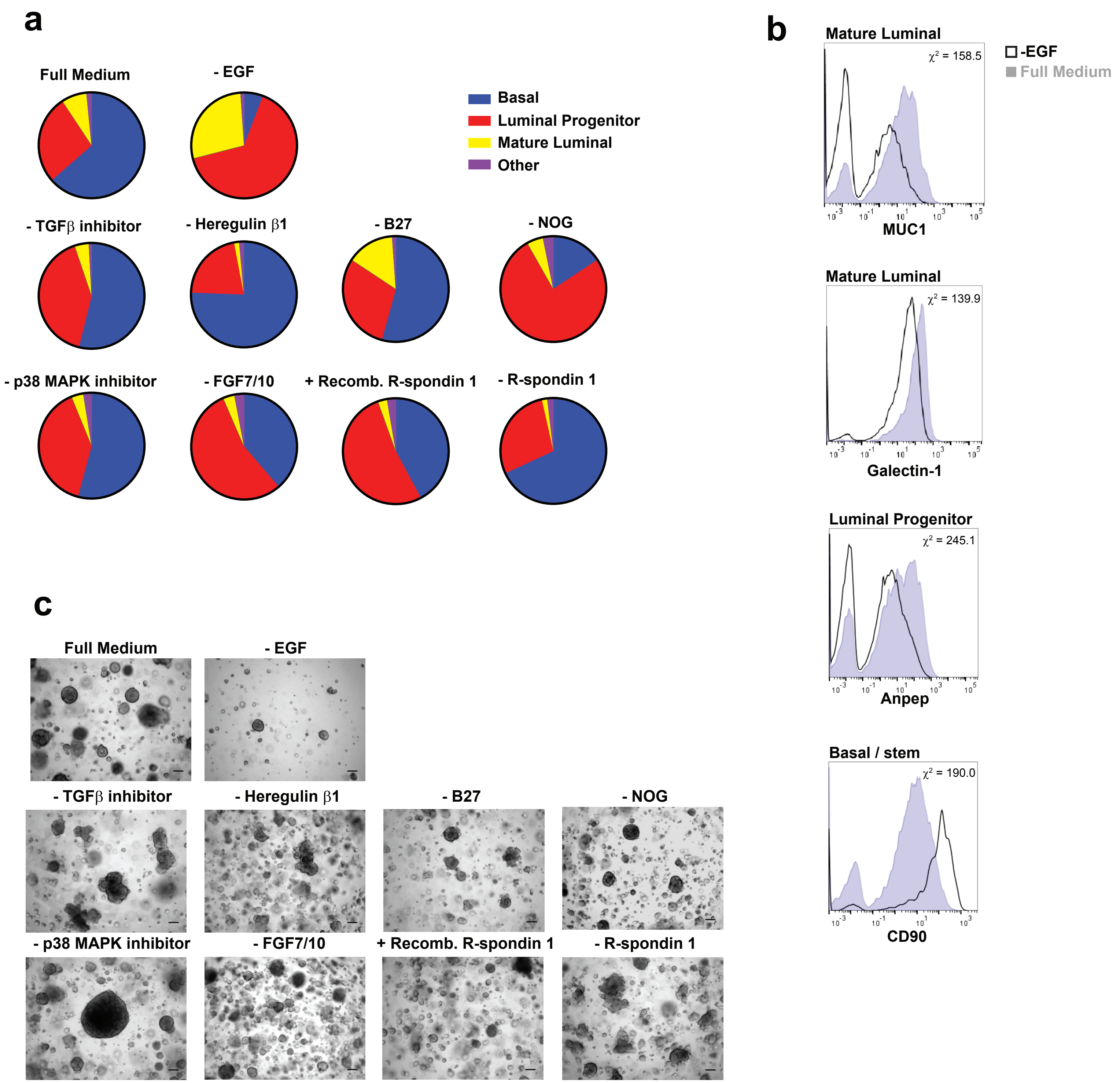

Supplementary Figure 12. Individual components of the organoid medium affect mammary lineage distribution in organoid cultures. For a representative mammary organoid culture, ORG63, organoids were established in the presence or absence of the indicated growth factors or signaling pathway inhibitors. **a** CyTOF analyses were performed on the series of cultures, and the percentages of cells in the mature luminal (ML), luminal progenitor (LP), and basal populations were determined, as shown by pie chart. **b** Marker expression levels are shown in the histograms as measured by CyTOF for mature luminal, luminal progenitor, or basal/stem cell populations of ORG63, as indicated. Shaded histogram indicates culture grown in full medium, and clear histogram indicates culture grown in the absence of EGF. χ^2 values shown. **c** Bright field images at low power of organoid cultures grown in full medium, medium with recombinant R-spondin 1 (control for - R-spondin 1 condition), or medium lacking the indicated factor (representative of n = 4). Scale bar = 100 μm.

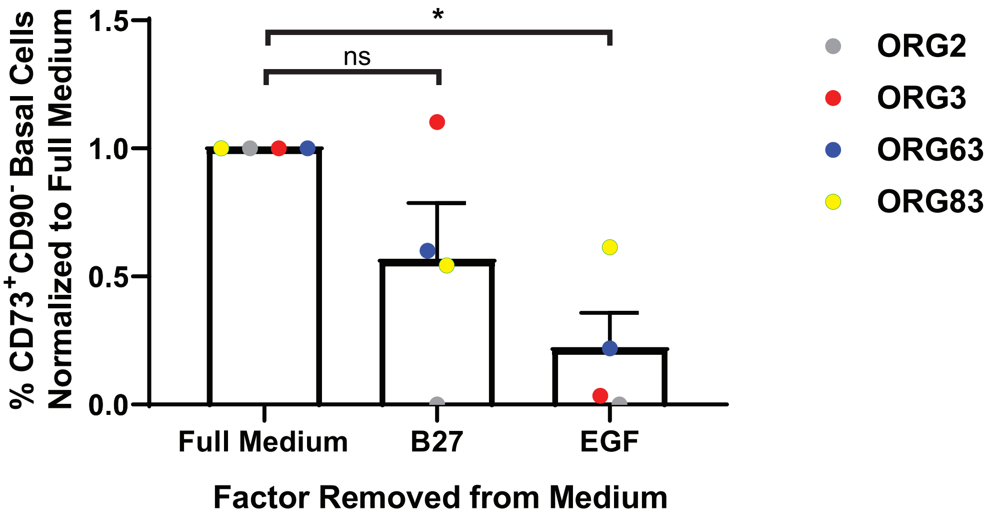

Supplementary Figure 13. Assessment of a $CD73^{+}$ $CD90^{-}$ population in organoid cultures established in full medium, or medium lacking either B27 or EGF. The percentage of $CD73^{+}$ $CD90^{-}$ cells in the basal ($EpCAM^{-}$ $CD49f^{+}$) population was determined as measured by CyTOF for the indicated cultures, values shown are normalized to the percentage of $CD73^{+}$ $CD90^{-}$ cells in the basal population grown in full medium. Mean and standard error of the mean are shown. * is $p = 0.012$ and ns is not significant by Student's t-test, two-tailed.

Supplementary Table 1

Assessment of branching or budding-type organoids		
**Culture**	**Number of branching/ budding organoids**	**Number of total organoids**
ORG3	6	479
ORG63	3	346
ORG83	5	553

Supplementary Table 2

Numbers of ER+ organoids and cells in culture				
Culture	Number of ER+ organoids	Number of total organoids	Number of ER+ cells	Number of total cells
ORG23	13	53	22	743
ORG9	40	70	66	217
ORG60	24	81	53	477
ORG63	45	244	239	3692
ORG66	67	162	7	758

Supplementary Table 3

Calculation of organoid forming efficiency						
	Mature Luminal		Luminal Progenitor		Basal / Stem	
Culture	Organoid Forming Efficiency	Number of sorted cells	Organoid Forming Efficiency	Number of sorted cells	Organoid Forming Efficiency	Number of sorted cells
ORG7	1 in 4	19579	1 in 11	33485	1 in 302	10571
ORG46	1 in 9	5239	1 in 14	22377	0 in 241	241
ORG41	1 in 9	1201	1 in 14	9354	0 in 24	24
ORG43	1 in 50	495	1 in 3	3760	1 in 16	334
ORG60	1 in 9	14717	1 in 8	8200	1 in 34	57370

Supplementary Table 4

Antibodies used for mass cytometry					
**Marker**	**Company**	**Catalog no.**	**Origin**	**Location**	**Metal**
CK8	DSHB	TROMA	rat	intracellular	145Nd
BRCA1	from D. Livingston	MS110	mouse	intracellular	169Tm
TSPAN8	Biolegend	363702	mouse	extracellular	169Tm
CD47	Fluidigm	3209004B	mouse	extracellular	209Bi
CD49f	Biolegend	313602	mouse	extracellular	155Gd
vimentin	Cell signaling	5741	rabbit	intracellular	174Yb
AR	Cell signaling	5153	rabbit	intracellular	143Nd
HER2	Cell signaling	2165	rabbit	intracellular	176Yb
GR	Cell signaling	3660	rabbit	intracellular	156Gd
PR B	Cell signaling	3157	rabbit	intracellular	164Dy
p53	Cell signaling	2524	mouse	intracellular	165Ho
CD95	Miltenyi Biotec	130-108-066	mouse 1gG1k	extracellular	168Er
CD133	Miltenyi Biotec	130-108-062	mouse IgG1k	extracellular	173Yb
GATA3	Miltenyi Biotec	130-108-061	human IgG1	intracellular	141Pr
Ki67	Miltenyi Biotec	130-108-060	human IgG1	intracellular	146Nd
CD45	Fluidigm	3089005B	human IgG1	extracellular	089Y
CD31	Biolegend	303102	mouse IgG1, k	extracellular	113In
ER a	Cell signaling	13258	rabbit	intracellular	172Yb
Parp (cleaved)	ebioscience	14-6668-80	mouse	intracellular	159Tb
SMA	ebioscience	14-9760-80	mouse	intracellular	160Gd
EpCAM	Biolegend	324202	mouse	extracellular	150Nd
CD24	Biolegend	311102	mouse	extracellular	158Gd
EPCR	Biolegend	351902	rat	extracellular	148Nd
MUC1	Biolegend	355602	mouse IgG1	extracellular	149Sm
LAM5	DSHB	P3H9	mouse	intracellular	162Dy
HSP27	DSHB	CPTC-HSPB1-3	mouse IgG2b	intracellular	153Eu
ANXA8	R & D Systems	AF8105-SP	sheep	intracellular	161Dy
Galectin-1	R & D Systems	AF1152-SP	goat	intracellular	171Yb
CK14	R & D Systems	MAB3164-SP	mouse IgG2a	intracellular	144Nd
HLA-ABC	Biolegend	311402	mouse	intracellular	167Er
CD10	Biolegend	312202	mouse	extracellular	163Dy
CD44	Biolegend	103001	rat	extracellular	115In
CD73	Biolegend	344002	mouse	extracellular	170Er
CD90	Biolegend	328101	mouse	extracellular	152Sm
ANPEP	Biolegend	301701	mouse	extracellular	151Eu
CD54	Biolegend	353101	mouse	extracellular	142Nd
EGFR	Biolegend	352901	mouse	extracellular	147Sm
CK17	Cell signaling	12509	rabbit	intracellular	166Er
RANK	Amgen	N-1H8	mouse	extracellular	175Lu
H3K27Me3	Cell signaling	9733S	rabbit	intracellular	175Lu
CD140b	Cell signaling	4564	rabbit	intracellular	154Sm
