# Supplementary material for: Organoid cultures from normal and cancer-prone human breast tissues preserve complex epithelial lineages
Source: Nat Commun. 2020 Apr 6;11:1711. doi: 10.1038/s41467-020-15548-7 (PMC7136203; doi:10.1038/s41467-020-15548-7)
Supplement: Supplementary file 2 — Description of Additional Supplementary Files [file 41467_2020_15548_MOESM2_ESM.docx]

**Description of Additional Supplementary Files**

File Name: Supplementary Data 1
Description: List and annotation of organoid cultures generated
