# Supplementary material for: Organoid cultures from normal and cancer-prone human breast tissues preserve complex epithelial lineages
Source: Nat Commun. 2020 Apr 6;11:1711. doi: 10.1038/s41467-020-15548-7 (PMC7136203; doi:10.1038/s41467-020-15548-7)
Supplement: Supplementary file 4 — Reporting Summary [file 41467_2020_15548_MOESM4_ESM.pdf]

nature research

Corresponding author(s): Joan S. Brugge

Last updated by author(s): 03/02/2020

Reporting Summary

Nature Research wishes to improve the reproducibility of the work that we publish. This form provides structure for consistency and transparency in reporting. For further information on Nature Research policies, see Authors & Referees and the Editorial Policy Checklist .

Statistics

For all statistical analyses, confirm that the following items are present in the figure legend, table legend, main text, or Methods section.

n/a	Confirmed	
☐	☒	The exact sample size (*n*) for each experimental group/condition, given as a discrete number and unit of measurement
☐	☒	A statement on whether measurements were taken from distinct samples or whether the same sample was measured repeatedly
☐	☒	The statistical test(s) used AND whether they are one- or two-sided<br>*Only common tests should be described solely by name; describe more complex techniques in the Methods section.*
☐	☒	A description of all covariates tested
☐	☒	A description of any assumptions or corrections, such as tests of normality and adjustment for multiple comparisons
☐	☒	A full description of the statistical parameters including central tendency (e.g. means) or other basic estimates (e.g. regression coefficient) AND variation (e.g. standard deviation) or associated estimates of uncertainty (e.g. confidence intervals)
☐	☒	For null hypothesis testing, the test statistic (e.g. *F*, *t*, *r*) with confidence intervals, effect sizes, degrees of freedom and *P* value noted<br>*Give P values as exact values whenever suitable.*
☐	☒	For Bayesian analysis, information on the choice of priors and Markov chain Monte Carlo settings
☐	☒	For hierarchical and complex designs, identification of the appropriate level for tests and full reporting of outcomes
☐	☒	Estimates of effect sizes (e.g. Cohen's *d*, Pearson's *r*), indicating how they were calculated

Our web collection on statistics for biologists contains articles on many of the points above.

Software and code

Policy information about availability of computer code

Data collection	No software was used
Data analysis	Heat maps were generated in R 3.5.1. Custom code has been deposited in GitHub (https://github.com/lselfors/Rosenbluth_organoid_cultures_NatureComm2020) and is also available upon request. The X-shift algorithm in the Vortex software package was used to analyze CyTOF data. Pearson's r was calculated in R 3.5.1. Statistical significance was assessed by Mann-Whitney test in JMP Pro 14.0.0.

For manuscripts utilizing custom algorithms or software that are central to the research but not yet described in published literature, software must be made available to editors/reviewers. We strongly encourage code deposition in a community repository (e.g. GitHub). See the Nature Research guidelines for submitting code & software for further information.

Data

Policy information about availability of data

All manuscripts must include a data availability statement. This statement should provide the following information, where applicable:
- Accession codes, unique identifiers, or web links for publicly available datasets
- A list of figures that have associated raw data
- A description of any restrictions on data availability

Raw CyTOF data for the organoids analyzed has been deposited in the publicly available database FlowRepository (www.flowrepository.org), under the following Repository IDs: FR-FCM-Z2HY (Figures 3, S4, S5, S6, S9), FR-FCM-Z2H2 (Figures 4, 5, S7), and FR-FCM-Z2H5 (Figures 8, S12, S13).

Field-specific reporting

Please select the one below that is the best fit for your research. If you are not sure, read the appropriate sections before making your selection.

- [x] Life sciences
- [ ] Behavioural & social sciences
- [ ] Ecological, evolutionary & environmental sciences

For a reference copy of the document with all sections, see nature.com/documents/nr-reporting-summary-flat.pdf

Life sciences study design

All studies must disclose on these points even when the disclosure is negative.

Sample size	Sample size for assessment of the percentage of luminal progenitor cells in organoid cultures with mutations in BRCA1, as compared to those without mutations, was determined by performing a pilot study with 8 samples. Sample size was extended to 24 based on power calculation.
Data exclusions	An organoid culture with its matching mammary tissue and HMEC culture was excluded. The barcoding failed prior to CyTOF analysis, resulting in a large number of unbarcoded cells and too few barcoded cells for analysis.
Replication	The reproducibility of the experiments was confirmed using at least three biological replicates or replicates involving multiple organoid cultures derived from different patients, as follows: Fig 1a representative of 79 organoid cultures, Fig 1b representative of 4 cultures, Fi 1c representative of 4 cultures, Fig 1d representative of 4 organoid cultures, Fig 1e n = 4 organoid cultures, Fig 1f n = 3 independent eexperiments, Fig 1g n = 3 organoid cultures are shown. Fig 2a multiple structure types representative of 79 organoid cultures, Fig 2b structure types representative of those seen in 79 organoid cultures, Fig 2c 9 organoid cultures were sorted as shown and representative examples of the analyses for these cultures are shown in Fig 2c, Fig 2d, and Fig 2e, Fig 2f representative of 6 organoid cultures, Fig 2g the one organoid culture shown was double-sorted as a control. Figures 3, S4, S5, S6, and S9 n = 12 organoid cultures were analyzed. Figures 4 and S7 n = 4 organoid cultures and n = 1 HMEC culture were analyzed. Fig 5 n = 1 tissue was processed to generate both an organoid culture and an HMEC culture, related to Fig 4 but with both culture types derived from the same tissue. Fig 6a n = 15 organoid cultures were analyzed, Fig 6b n = 12 organoid cultures were analyzed, Fig 6c n = 5 organoid cultures and matching tissues were analyzed. Figures 7 and S10 n = 12 wild-type and n = 12 BRCA1 heterozygous cultures were analyzed. Figures 8, S12, and S13 n = 4 organoid cultures were analyzed. Figure S11 n = 2 organoid cultures were of the type "mature luminal-cell predominant".
Randomization	Randomized studies were not included as organoid cultures were generated from all reduction mammoplasty and prophylactic mastectomy tissues that were obtained and as possible (e.g. a researcher was available to collect and process the tissue). In this study there were no drug or other treatments of samples within one group where it would be necessary to randomize samples to receive the treatment or not.
Blinding	All but one of the experiments involved characterization of lineages preserved in organoid cultures and were not a comparison of one genotype or treatment type to another; this also minimizes the potential for bias due to lack of blinding. In the one experiment the % of LP cells were compared in samples carrying WT and mutant BRCA. In the CyTOF experiments, the individual preparing and running the samples did not have access to the master key that indicated the genotypes of the different samples. However, by necessity the individual on the team who reported the distribution of cell lineages did have to access to the key regarding the identity of the samples. Steps were taken to prevent bias including naming the organoid cultures by a strictly numerical system that does not indicate the genotype of the sample (e.g. ORG1, ORG2, ORG3...).

Reporting for specific materials, systems and methods

We require information from authors about some types of materials, experimental systems and methods used in many studies. Here, indicate whether each material, system or method listed is relevant to your study. If you are not sure if a list item applies to your research, read the appropriate section before selecting a response.

Materials & experimental systems

n/a	Involved in the study
[ ]	[x] Antibodies
[x]	[ ] Eukaryotic cell lines
[x]	[ ] Palaeontology
[x]	[ ] Animals and other organisms
[ ]	[x] Human research participants
[x]	[ ] Clinical data

Methods

n/a	Involved in the study
[x]	[ ] ChIP-seq
[ ]	[x] Flow cytometry
[x]	[ ] MRI-based neuroimaging

Antibodies

Antibodies used	The following primary antibodies were used: rabbit polyclonal anti-cleaved caspase 3 (Cell Signaling, Cat. No. 9661L), rabbit monoclonal anti-cytokeratin 8 (Alexa Fluor 488) (Abcam, Cat. No. ab192467), mouse monoclonal anti-cytokeratin 8 (Santa Cruz, sc-8020), rabbit monoclonal anti-cytokeratin 14 (Alexa Fluor 647) (Abcam, Cat. No. ab206100), rabbit polyclonal anti-cytokeratin 14 (BioLegend, Cat. No. 905301), rabbit monoclonal anti-cytokeratin 19 (Abcam, ab52625), rabbit monoclonal anti-Estrogen Receptor α (Abcam, Cat. No. ab16660), mouse monoclonal anti-EpCAM (Alexa Fluor 647) (Biolegend, Cat. No. 324212), rat

monoclonal anti-CD49f (phycoerythrin) (Biolegend, Cat. No. 313611), rabbit monoclonal anti-ERBB3 (Abcam, ab236220), and rabbit polyclonal anti-Ki67 (Abcam, Cat. No. ab15580). The following antibodies were used for CyTOF with the company and catalog number indicated in parentheses or an indication of the source of the antibody: CK8 (DSHB, TROMA), BRCA1 (from D. Livingston, MS110), TSPAN8 (Biolegend, 363702), CD47 (Fluidigm, 3209004B), CD49f (Biolegend, 313602), vimentin (Cell signaling, 5741), AR (Cell signaling, 5153), HER2 (Cell signaling, 2165), GR (Cell signaling, 3660), PR B (Cell signaling, 3157), p53 (Cell signaling, 2524), CD95 (Miltenyi Biotec, 130-108-066), CD133 (Miltenyi Biotec, 130-108-062), GATA3 (Miltenyi Biotec, 130-108-061), Ki67 (Miltenyi Biotec, 130-108-060), CD45 (Fluidigm, 3089005B), CD31 (Biolegend, 303102), ER a (Cell signaling, 13258), Parp (cleaved) (ebioscience, 14-6668-80), SMA (ebioscience, 14-9760-80), EpCAM (Biolegend, 324202), CD24 (Biolegend, 311102), EPCR (Biolegend, 351902), MUC1 (Biolegend, 355602), LAM5 (DSHB, P3H9), HSP27 (DSHB, CPTC-HSPB1-3), ANXA8 (R & D Systems, AF8105-SP), Galectin-1 (R & D Systems, AF1152-SP), CK14 (R & D Systems, MAB3164-SP), HLA-ABC (Biolegend, 311402), CD10 (Biolegend, 312202), CD44 (Biolegend, 103001), CD73 (Biolegend, 344002), CD90 (Biolegend, 328101), ANPEP (Biolegend, 301701), CD54 (Biolegend, 353101), EGFR (Biolegend, 352901), CK17 (Cell signaling, 12509), RANK (Amgen, N-1H8),. H3K27Me3 (Cell signaling, 9733S), and CD140b (Cell signaling, 4564).

Validation

The following primary antibodies were used: Rabbit polyclonal anti-cleaved caspase 3 (Cell Signaling, Cat. No. 9661L) has been validated by immunohistochemistry and immunofluorescence as indicated on the manufacturer's website, and has been used previously by our laboratory1. Rabbit monoclonal anti-cytokeratin 8 (Alexa Fluor 488) (Abcam, Cat. No. ab192467) was validated by confirming tissue staining in luminal mammary epithelial cells in tissue samples, but absence of staining in appropriate basal and stromal cells by immunofluorescence. Mouse monoclonal anti-cytokeratin 8 (Santa Cruz, sc-8020) was validated by confirming immunostaining in luminal epithelial cells in breast tissues, but absence of staining in the appropriate basal epithelial and stromal cells. Rabbit monoclonal anti-cytokeratin 14 (Alexa Fluor 647) (Abcam, Cat. No. ab206100) was validated by confirming immunostaining in basal epithelial cells in breast tissues, but absence of staining in the appropriate stromal compartments. Rabbit polyclonal anti-cytokeratin 14 (BioLegend, Cat. No. 905301) was validated by confirming immunostaining in basal epithelial cells in breast tissues, but absence of staining in the appropriate stromal compartments. Rabbit monoclonal anti-cytokeratin 19 (Abcam, ab52625) was validated by confirming immunostaining in the luminal epithelial cells in breast tissues, but absence of staining in the appropriate basal epithelial and stromal cells. Rabbit monoclonal anti-Estrogen Receptor α (Abcam, Cat. No. ab16660) is noted on the manufacturer's website to be validated for assays including immunohistochemistry and immunofluorescence and tested in human. Validated by confirming nuclear immunostaining in luminal mammary epithelial cells. Mouse monoclonal anti-EpCAM (Alexa Fluor 647) (Biolegend, Cat. No. 324212) is noted on the manufacturer's website to be validated for immunofluorescence and quality tested for flow cytometry. This antibody was further validated for flow cytometry by inspection of biaxial plots for expected staining patterns of mammary epithelial cells. Rat monoclonal anti-CD49f (phycoerythrin) (Biolegend, Cat. No. 313611) is noted to be quality tested on the manufacturer's website. This antibody was validated for flow cytometry by inspection of biaxial plots for expected staining patterns of mammary epithelial cells. Rabbit monoclonal anti-ERBB3 (Abcam, ab236220) is noted to be guaranteed for immunohistochemistry on formalin-fixed paraffin-embedded sections on the manufacturer's website. Rabbit polyclonal anti-Ki67 (Abcam, Cat. No. ab15580) is noted to have been validated on the manufacturer's website by performing immunostaining in HAP1 cells with and without knock-out of Ki67, and has also been validated for immunohistochemistry on formalin-fixed and paraffin-embedded sections. Validation was also performed by inspection for nuclear signal. CK8 (DSHB, TROMA) was validated by performing flow cytometry on breast and other cell lines (e.g. signal in MCF-7 cells) and inspection of biaxial plots after performing mass cytometry on mammary cells. BRCA1 (from D. Livingston, MS110) was validated by performing immunohistochemistry on formalin-fixed paraffin embedded breast tissue, by performing flow cytometry on breast and other cell lines (e.g. signal in MCF-7 cells), and by performing mass cytometry on mammary cells by inspection of biaxial plots. TSPAN8 (Biolegend, 363702) was validated by performing flow cytometry on breast and other cell lines (e.g. signal in MDA-MB-231 cells), and by performing mass cytometry on mammary cells by inspection of biaxial plots. On the manufacturer's website it is noted to be quality tested for flow cytometry. CD47 (Fluidigm, 3209004B) was validated by performing flow cytometry on breast and other cell lines (e.g. signal in BT549 cells), and by performing mass cytometry on mammary cells by inspection of biaxial plots. On the manufactuerer's website it is noted to be quality tested for CyTOF. CD49f (Biolegend, 313602) was validated by performing flow cytometry on breast and other cell lines (e.g. signal in MDA-MB-468 cells), and by performing mass cytometry on mammary cells by inspection of biaxial plots. It is noted to be validated for immunocytochemistry and quality tested for flow cytometry on the manufacturer's website. Vimentin (Cell signaling, 5741) was validated by performing immunofluorescence on human breast and skin tissues, by performing flow cytometry on breast and other cell lines (e.g. signal in MDA-MB-231 cells), and by performing mass cytometry on mammary cells by inspection of biaxial plots. AR (Cell signaling, 5153) was validated by performing immunofluorescence on human breast tissues with signal seen in the nuclei of luminal epithelial cells, by performing flow cytometry on breast and other cell lines (e.g. signal in MDA-MB-453 cells), and by performing mass cytometry on mammary cells by inspection of biaxial plots. HER2 (Cell signaling, 2165) was validated by performing immunofluorescence on human breast ductal carcinoma in situ from a xenograft, by performing flow cytometry on breast and other cell lines (e.g. signal in BT474 cells), and by performing mass cytometry on mammary cells by inspection of biaxial plots. GR (Cell signaling, 3660) was validated by performing immunofluorescence on human breast tissue, by performing flow cytometry on breast and other cell lines (e.g. signal in BT549 cells), and by performing mass cytometry on mammary cells by inspection of biaxial plots. PR B (Cell signaling, 3157) was validated by performing immunofluorescence on human breast tissue, by performing flow cytometry on breast and other cell lines (e.g. signal in BT474 cells), and by performing mass cytometry on mammary cells by inspection of biaxial plots. p53 (Cell signaling, 2524) was validated by performing immunofluorescence on human fallopian tube tissue, by performing flow cytometry on breast and other cell lines (e.g. signal in MDA-MB-468 cells), and by performing mass cytometry on mammary cells by inspection of biaxial plots. CD95 (Miltenyi Biotec, 130-108-066) was validated by performing immunofluorescence on human breast tissue, by performing flow cytometry on breast and other cell lines (e.g. signal in MCF10A cells), and by performing mass cytometry on mammary cells by inspection of biaxial plots. CD133 (Miltenyi Biotec, 130-108-062) was validated by performing flow cytometry on breast and other cell lines (e.g. signal in MDA-MB-468 cells), and by performing mass cytometry on mammary cells by inspection of biaxial plots. GATA3 (Miltenyi Biotec, 130-108-061) was validated by performing flow cytometry on breast and other cell lines (e.g. signal in MCF-7 cells), and by performing mass cytometry on mammary cells by inspection of biaxial plots. Ki67 (Miltenyi Biotec, 130-108-060) was validated by performing flow cytometry on cell lines (e.g. signal in Raji cells), by performing immunofluorescence on human ductal carcinoma in situ cells in a xenograft, and by performing mass cytometry on mammary cells by inspection of biaxial plots. CD45 (Fluidigm, 3089005B) was validated by performing flow cytometry on cell lines (e.g. signal in Raji cells) and mass cytometry on mammary cells by inspection of biaxial plots, and on the manufacturer's website is noted to be quality control tested by CyTOF analysis.

CD31 (Biolegend, 303102) is noted to be quality tested for flow cytometry and validated for immunocytochemistry on the manufacturer's website. It was validated by performing mass cytometry on mammary cells by inspection of biaxial plots. ER a (Cell signaling, 13258) was validated by performing flow cytometry on breast and other cell lines (e.g. signal in MCF-7 and T47D cells), and by performing mass cytometry on mammary cells by inspection of biaxial plots. Parp (cleaved) (ebioscience, 14-6668-80) was validated by performing flow cytometry on cell lines (e.g. MM.1S cells) treated with staurosporine (Tocris, Cat. No. 1285), and by performing mass cytometry on mammary cells by inspection of biaxial plots. SMA (ebioscience, 14-9760-80) was validated by performing flow cytometry on cell lines (e.g. signal in NIH3T3 cells), and by performing mass cytometry on mammary cells by inspection of biaxial plots. EpCAM (Biolegend, 324202) was validated by performing immunofluorescence on human mammary tissue, by performing flow cytometry on breast and other cell lines (e.g. signal in MCF-7 cells), and by performing mass cytometry on mammary cells by inspection of biaxial plots. CD24 (Biolegend, 311102) was validated by performing flow cytometry on breast and other cell lines (e.g. signal in MDA-MB-468 cells), and by performing mass cytometry on mammary cells by inspection of biaxial plots. EPCR (Biolegend, 351902) was validated by performing flow cytometry on breast and other cell lines (e.g. signal in MDA-MB-157 cells), and by performing mass cytometry on mammary cells by inspection of biaxial plots. MUC1 (Biolegend, 355602) was validated by performing flow cytometry on breast and other cell lines (e.g. signal in MCF-7 cells), and by performing mass cytometry on mammary cells by inspection of biaxial plots. LAM5 (DSHB, P3H9) was validated by performing flow cytometry on breast and other cell lines (e.g. signal in HCC1937 cells), by performing immunohistochemistry on human tonsil tissue, and by performing mass cytometry on mammary cells by inspection of biaxial plots. HSP27 (DSHB, CPTC-HSPB1-3) was validated by performing immunohistochemistry on human skin tissue, by performing flow cytometry on breast and other cell lines (e.g. signal in MCF-7 cells), and by performing mass cytometry on mammary cells by inspection of biaxial plots. ANXA8 (R & D Systems, AF8105-SP) was validated by performing flow cytometry on breast and other cell lines (e.g. signal in quiescent MCF10A cells), and by performing mass cytometry on mammary cells by inspection of biaxial plots. Galectin-1 (R & D Systems, AF1152-SP) was validated by performing flow cytometry on breast and other cell lines (e.g. signal in HCC1937 cells), by performing mass cytometry on mammary cells by inspection of biaxial plots. CK14 (R & D Systems, MAB3164-SP) was validated by performing immunohistochemistry on human breast and tonsil tissues, by performing flow cytometry on breast and other cell lines (e.g. signal in HCC1937 cells), by performing mass cytometry on mammary cells by inspection of biaxial plots. HLA-ABC (Biolegend, 311402) was validated by performing flow cytometry on breast and other cell lines (e.g. signal in MDA-MB-157 cells), and by performing mass cytometry on mammary cells by inspection of biaxial plots. CD10 (Biolegend, 312202) was validated by performing flow cytometry on breast and other cell lines (e.g. signal in MDA-MB-157 cells), and by performing mass cytometry on mammary cells by inspection of biaxial plots. CD44 (Biolegend, 103001) was validated by performing immunohistochemistry on human breast tissues, by performing flow cytometry on breast and other cell lines (e.g. signal in MDA-MB-231 cells), and by performing mass cytometry on mammary cells by inspection of biaxial plots. CD73 (Biolegend, 344002) was validated by performing flow cytometry on breast and other cell lines (e.g. signal in MDA-MB-231 cells), and by performing mass cytometry on mammary cells by inspection of biaxial plots. CD90 (Biolegend, 328101) was validated by performing flow cytometry on breast and other cell lines (e.g. signal in BT549 cells), and by performing mass cytometry on mammary cells by inspection of biaxial plots. ANPEP (Biolegend, 301701) was validated by performing flow cytometry on breast and other cell lines (e.g. signal in MCF10A cells), and by performing mass cytometry on mammary cells by inspection of biaxial plots. CD54 (Biolegend, 353101) was validated by performing flow cytometry on breast and other cell lines (e.g. signal in MDA-MB-468 cells), and by performing mass cytometry on mammary cells by inspection of biaxial plots. EGFR (Biolegend, 352901) was validated by performing flow cytometry on breast and other cell lines (e.g. signal in MDA-MB-468 cells), and by performing mass cytometry on mammary cells by inspection of biaxial plots. CK17 (Cell signaling, 12509) was validated by performing flow cytometry on breast and other cell lines (e.g. signal in MDA-MB-468 cells), and by performing mass cytometry on mammary cells by inspection of biaxial plots. RANK (Amgen, N-1H8) was validated by performing mass cytometry on mammary organoid cells by inspection of biaxial plots. It has been used for flow cytometry on human breast cells previously2. H3K27Me3 (Cell signaling, 9733S) is noted on the manufacturer's website to have been validated using SimpleChIP® Enzymatic Chromatin IP Kits. It has been validated by performing flow cytometry (on HEK293T cells treated with and without GSK126 purchased from Selleckchem, Cat. No. S7061) and by mass cytometry on breast organoid cells by inspection of biaxial plots. CD140b (Cell signaling, 4564) was validated by performing flow cytometry on breast cells, and by performing mass cytometry on human breast cells with inspection of biaxial plots. It is shown on the manufacturer's website to stain lung tissue by immunohistochemistry.

References:

1 Zervantonakis, I. K. et al. Systems analysis of apoptotic priming in ovarian cancer identifies vulnerabilities and predictors of drug response. Nat Commun 8, 365, doi:10.1038/s41467-017-00263-7 (2017).

2 Nolan, E. et al. RANK ligand as a potential target for breast cancer prevention in BRCA1-mutation carriers. Nat Med 22, 933-939, doi:10.1038/nm.4118 (2016).

Human research participants

Policy information about studies involving human research participants

Population characteristics	Please see Supplementary Data for description relevant to patient-derived organoid cultures.
Recruitment	Breast tissues were obtained from reduction mammoplasty or prophylactic mastectomy samples at Brigham & Women's Hospital or Faulkner Hospital.
Ethics oversight	Harvard Medical School Institutional Review Board reviewed this study and deemed it not human subjects research.

Note that full information on the approval of the study protocol must also be provided in the manuscript.

Flow Cytometry

Plots

Confirm that:

- [x] The axis labels state the marker and fluorochrome used (e.g. CD4-FITC).
- [x] The axis scales are clearly visible. Include numbers along axes only for bottom left plot of group (a 'group' is an analysis of identical markers).
- [x] All plots are contour plots with outliers or pseudocolor plots.
- [x] A numerical value for number of cells or percentage (with statistics) is provided.

Methodology

Sample preparation	Single cells were obtained from organoid cultures as described in Methods.
Instrument	BD LSR Fortessa, with the assistance of the Dana Farber Flow Cytometry Core
Software	Data was collected with BD FACSDiva. Data was analyzed in FlowJo.
Cell population abundance	Abundance (percentage) is indicated for the relevant populations on flow cytometry plots.
Gating strategy	Cells are first identified using FSC/SSC, then epithelial cells followed by mammary epithelial lineages are identified using EpCAM and CD49f. Figure 2c exemplifies the gating used for FACS.

- [x] Tick this box to confirm that a figure exemplifying the gating strategy is provided in the Supplementary Information.
